# Causal Networks to Inform Decisions for Ecological Restoration

**DOI:** 10.1007/s00267-025-02323-x

**Published:** 2025-11-20

**Authors:** Christopher J. Kotalik, Freya E. Rowland, Bruce G. Marcot, Kristin E. Skrabis, David M. Walters, Jo Ellen Hinck, William H. Clements, Eric E. Richer, John P. Isanhart

**Affiliations:** 1 U.S. Geological Survey, Columbia Environmental Research Center, Columbia, MO USA; 2 U.S. Forest Service, Pacific Northwest Research Station, Portland, OR USA; 3 U.S. Department of the Interior, Office of Policy Analysis, Washington, DC USA; 4 U.S. Geological Survey, Natural Hazards Mission Area, Reston, VA USA; 5 Department of Fish, Wildlife, and Conservation Biology, Colorado State University, Fort Collins, CO USA; 6 Aquatic Research Section, Colorado Parks and Wildlife, Fort Collins, CO USA; 7 U.S. Department of the Interior, Office of Restoration and Damage Assessment, Denver, CO USA

**Keywords:** Causal network, Habitat restoration, Natural Resource Damage Assessment and Restoration, Decision advisory tool

## Abstract

The release of contaminants into the environment can occur from anthropogenic activities, such as oil extraction and transportation, mining, and industrial processes. Remediation associated with reducing contaminant concentrations, and restoration that improves animals and supporting habitat, are often needed to restore ecosystems to their pre-release, baseline condition. We demonstrated the application of Bayesian Decision Networks (BDNs) with two Natural Resource Damage Assessment and Restoration (NRDAR) case studies. We use a stylized case study of riparian restoration following the remediation of a mine-impacted site to evaluate proposed restoration actions aimed at restoring Song Sparrow (*Melospiza melodia*) populations to baseline conditions. We then use a settled NRDAR case with implemented restoration in the Upper Arkansas River (UAR, Colorado, USA) to demonstrate the application of BDNs to evaluate and forecast restoration effectiveness for Brown Trout (*Salmo trutta*) (i.e., restoration effectiveness assessment). The riparian restoration model showed differences in the effects of restoration actions on Song Sparrow populations, with the time to reach baseline generally reduced with increased restoration costs, indicating trade-offs between costs and expected recovery. The UAR model showed recovery of Brown Trout populations (i.e., uplift) in response to improved instream habitat restoration, along with forecasted improvements. While the BDNs we developed were specific to two case studies, the structure is adaptable to a diversity of sites, resources, and actions. We suggest that causal network modeling can provide restoration practitioners with a decision advisory tool useful for a wide range of projects.

## Introduction

The release of contaminants into the environment occurs from anthropogenic activities, such as oil extraction and transportation, mining, and industrial processes. Direct and indirect effects on natural resources (NR) can vary based on the contaminant (e.g., metals vs persistent organic contaminants), frequency and magnitude of release (e.g., oil spill incident vs chronic acid mine drainage), and the ecosystem affected (Fleeger et al., 2003; Clements et al., 2012; Krithiga et al., 2022). Remediation associated with reducing contaminant concentrations, and actions that restore animal populations and improve habitat (e.g., restoration, mitigation, reclamation, collectively referred to as restoration in this paper), are often needed to return ecosystems to pre-release, baseline condition (Clements et al., 2021). Practitioners are typically confronted with complex ecological systems at contaminated sites and tasked with determining the next best steps given the available budget. There is a recognized need among restoration practitioners for more decision advisory tools and supporting analyzes. Our goal was to address this need by providing a potential framework for more fully expressing actions and results of restoration decision processes. The framework is illustrated using Natural Resource Damage Assessment and Restoration (NRDAR) case studies.

**Fig. 1:**
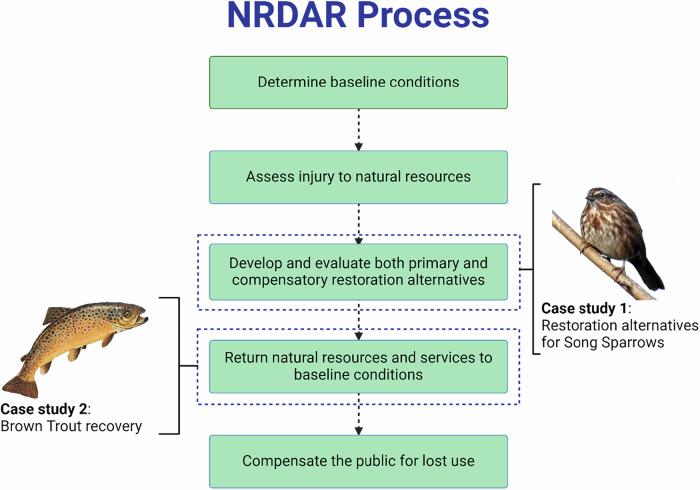
The Natural Resource Damage Assessment and Restoration (NRDAR) process for restoring injured ecosystems to baseline conditions (see glossary for definitions). We demonstrate how Bayesian decision networks can help explore proposed restoration actions using a stylized case study of Song Sparrows (*Melospiza melodia*; photo credit B. Marcot) and in evaluating restoration effectiveness using a case study of Brown Trout recovery (*Salmo trutta*; photo credit Great Lakes Environmental Research Laboratory, National Oceanic and Atmospheric Administration, public domain) following restoration of the Upper Arkansas River, Colorado, USA

The U.S. Department of the Interior NRDAR Program restores natural resources that have been injured by the release of oil or other hazardous substances into the environment (as defined in 43 CFR §11.64 and 15 CFR § 990). Environmental “injury” is quantified as a measurable change in the chemical or physical viability of a natural resource resulting directly or indirectly from contaminant release (43 CFR § 11.14). Injury assessment can include animals (e.g., fish, birds, turtles, and bats), their habitats (e.g., wetlands, forests, or streams), and ecosystem services provided by those animals and habitats. Once the NR injury has been determined, economists quantify the injuries, often using methods such as habitat equivalency analysis or resource equivalency analysis (REA) (Baker et al., 2020; Kraus et al., 2023; Table 1). Authorized representatives from federal (40 CFR § 300.600), state (CFR § 300.605), or Tribal governments (CFR § 300.610), referred to as Natural Resource Trustees (hereafter, Trustees), explore restoration actions with the goal of returning the natural resources and services to baseline conditions. Here, the baseline refers to the condition of natural resources, characterized by the absence of contamination, while also accounting for confounding factors such as development, agriculture, and climate change that may affect the baseline (Moe et al., 2024; Rohr et al., 2013, Fig. 1, Table 1). The goal of the NRDAR process is to determine the type and extent of restoration actions necessary to restore natural resources to their baseline conditions and replace those that were lost while these resources were impaired (Fig. 1). Trustees are tasked with developing and implementing a plan for the restoration, rehabilitation, replacement, or acquisition of the equivalent of the natural resources under their trusteeship (43 CFR 11.93). Trustees evaluate restoration actions based on many criteria, including the likelihood of success, prevention of future injury or collateral injury, benefits to more than one resource and/or service, effects on public health and safety, and environmental compliance, among others. Proposed restoration actions must also demonstrate a nexus to the injury. The extent of restoration credits produced from a habitat equivalency analysis or REA (Baker et al., 2020) depends on the rate of “uplift” (Table 1) from the proposed restoration over time (e.g., vegetation growth, animal density), and projected total time to return to baseline. NRDAR is not punitive, as Trustees are obligated to select the most cost-effective restoration action(s) (i.e., restoration goals are met at the least cost).

**Table 1:** Glossary of terms

Term	Definition	Example(s)
Baseline	The condition(s) that would have existed had the release of hazardous substances not occurred (43 CFR § 11.14). Another way to phrase it is “but for” the contamination, what would be the past, present, and future condition of the resource?	How many Song Sparrows (*Melospiza melodia*) would be living in a riparian area if there had not been mining tailings deposited on the stream bank? Baseline could be determined by quantifying Song Sparrow densities in a representative upstream reach without mining disturbance.
Discounting	Where possible, damages are estimated using an expected present value dollar amount, and a discounting rate is applied to the time it takes to achieve restoration (43 CFR § 11.84; Julius, 1999). Discounting can be viewed as a social rate of time preference (Horsch et al., 2023). It reflects society’s “impatience”, i.e., a bird in the future is worth less than one in the present or in the past.	The discount factor for each year is calculated as 1/(1+*r*)^time^. Time is relative to the base year; *r* is the discount rate of 3%. Future bird years are worth less than bird years at present. Because of discounting, it is prudent to address restoration as soon as possible.
Discounted bird-years (DBYs)	The count or density of birds injured over time is expressed as discounted bird-years (DBYs). DBYs account for the difference between the baseline and the injured resources, as well as the time required to come back to baseline. A discount rate is applied to convert all past and future losses and uplift from restoration actions into present value.	Loss of Song Sparrow density can be calculated in terms of discounted bird years based on degradation of riparian habitat and mercury exposure (the injury), which can be compensated for through restoration intended to re-establish native riparian vegetation, thus creating suitable habitat and potentially increasing Song Sparrow densities (restoration).
Habitat Equivalency Analysis (HEA)	Quantification of ecological service losses due to contamination relative to those gained through restoration, typically expressed as discounted service acre-years (DSAYS; Baker et al., 2020)	A DSAY is the present value of the total services provided by one unit of habitat over one year (Baker et al., 2020). For example, the ecological services provided by one hectare of wetland could include the food and habitat for the biota that live there as well as nutrient sequestration.
Natural resources	Natural resources include land, fish, wildlife, biota, air, water, ground water, drinking water supplies, and other resources belonging to or held in trust by the United States, and State or local government, any Tribe, or foreign government (43 CFR § 11.14).	The Trustees identify the relevant injured natural resources within their jurisdiction for assessment. For example, the Song Sparrow is a migratory bird, which is a U.S. Department of Interior-managed resource.
Natural resource injury	Measurable adverse change in the chemical or physical viability of a natural resource resulting directly or indirectly from a discharge of oil or release of a hazardous substance (43 CFR § 11.14).	Concentrations and release duration of contaminants exceed water quality criteria; concentrations of polychlorinated biphenyls in fish are sufficient to cause behavioral abnormalities, physical deformities, or death.
Remediation	The cleanup and reduction of hazardous substances, which is often authorized under CERCLA, typically occurs before restoration can take place.	Remediation may involve dredging contaminated sediments. Trustees typically consider the effects of remediation on injured natural resources over time.
Resource equivalency analysis (REA)	A REA is similar to a HEA but applies to living organisms (e.g., birds, mussels, turtles, bats) rather than spatial units.	A common unit of measure is the number of animals killed or the occurrence of sublethal effects, such as reduced reproduction (Baker et al., 2020).
Restoration	Actions taken to return an injured resource to the baseline physical, chemical, or biological services (43 CFR § 11.14). It can occur after or concurrently with remediation.	Restoration can include actions such as plantings, bank stabilization, soil amendments, controlled burns, removal of invasive species, or the installation of instream habitat structures.
Uplift	Habitat credits provided by restoration relative to baseline, accounting for any natural recovery (Baker et al., 2020)	Stream habitat restoration increases Brown Trout (*Salmo trutta*) biomass relative to baseline conditions, considering the initial conditions of the restoration location.

Natural Resource Damage Assessment and Restoration (NRDAR) regulations are outlined in the Comprehensive Environmental Response, Compensation and Liability Act of 1980, 42 USC §9601, et seq. (CERCLA) and the Oil Pollution Act of 1990, 33 USC. §2701, et seq. (OPA). The NRDAR regulations are defined in 43 CFR Part 11. The economic aspects of NRDAR cases are well-defined elsewhere (e.g., Baker et al. 2020); however, we provide some context for the NRDAR-specific definitions used throughout this paper

There are many technical challenges in planning and assessing the effectiveness of restoration, including the complex interpretation of statistically measured outcomes and difficulty in defining the temporal and spatial scales of ecological responses to injury and restoration. Several advisory frameworks have been developed to assist practitioners in evaluating NRDAR injury and economic damages, including an aquatic-terrestrial assessment tool (Kraus et al., 2023), a decision support framework for establishing restoration goals (Wagner et al., 2016), and the Habitat-based Resource Equivalency Method, which helps quantify habitat restoration for multiple injured resources (Baker et al., 2020). Rowland et al. (2024) illustrated how Bayesian networks (BNs) can integrate different types of data (e.g., empirical, toxicity benchmarks, expert knowledge) that incorporate direct and intermediate effects of contaminant exposure to estimate injury probability. Here, we extend the use of BNs to show how Bayesian decision networks (BDNs), which include decision and utility nodes, can inform restoration planning and evaluate restoration effectiveness by integrating ecological and economic information in a unified framework.

Our objective is to demonstrate the application of BDNs in two phases of NRDAR restoration in mining-impacted watersheds: (1) evaluation of potential restoration actions used for settlement; and (2) effectiveness assessment of implemented restoration, which could be used to inform adaptive management, as well as future NRDAR cases with similar contamination and natural resource injuries. We first use a stylized case study of riparian restoration following remediation of a mine-impacted site to demonstrate the application of BDNs for evaluating proposed restoration actions aimed at restoring Song Sparrow (*Melospiza melodia*) populations to baseline conditions. We then use a settled NRDAR case with implemented restoration to demonstrate the application of BDNs in evaluating restoration effectiveness, including forecasting responses of Brown Trout (*Salmo trutta*) to instream habitat restoration (i.e., restoration effectiveness assessment). In this study, we describe the methods used to construct and interpret BDNs and discuss the utility of BDNs as decision-advisory tools specifically for NRDAR cases and restoration practitioners more broadly.

## Methods

### Use of the Probability Network Approach

We used causal networks denoting the influence of restoration management decisions on environmental conditions. Specifically, we used BNs as the modeling structure (Koski and Noble, 2011). BNs are directed acyclic graphs that link variables (nodes) with conditional probabilities using Bayes’ theorem (Niedermayer, 2008). BNs are widely used in contexts of ecological studies and natural resource management (e.g., Landis et al. 2020), including areas of ecotoxicology (Carriger and Barron, 2020; Howes et al., 2010; Moe et al., 2021; Rowland et al., 2024), to depict and predict responses of species to environmental conditions and management activities.

Variables in BNs can be continuous, ordinal, categorical, structured as constants, or with conditional equations. Variables are typically denoted by two or more states, each with an associated probability. Conditional probability tables of each variable can represent empirically observed frequencies of outcome states of the variable, or as uncertainty of outcome states if the probabilities are spread across two or more states for a particular set of prior conditions. BNs, specifically BDNs, also include explicit decision nodes that depict alternative management decision actions and (optional) utility nodes that represent the outcomes (e.g., benefits, costs). The decision nodes depict the expected values of each set of restoration actions, which are the utility functions as weighted by the underlying probability structure of the network. Utility nodes can be structured as monetary cost functions, or, unlike the stricter structure of decision trees, as a mix of other units of measure denoting costs and/or benefits of outcomes. We developed two case-study BDN models: one to explore pre-settlement restoration planning and a model to evaluate restoration effectiveness.

### Restoration Planning: A Stylized Case Study of Riparian Restoration

We use a stylized example of an NRDAR case study to illustrate how to develop a BDN in the restoration planning process to inform settlement. This example involves an abandoned mercury mine site in the western United States, where the primary contaminant of concern is mercury (Hg) found predominantly in tailing piles deposited in a riparian corridor. Remediation removed ~50,000 cubic meters of tailings and reduced Hg exposure to bird species in the riparian zone. The Trustees decided to evaluate injury (defined in Table 1) to the Song Sparrow (*M. melodia*), an obligate riparian species. The site has insufficient riparian vegetation for nesting and to protect the Song Sparrow from predation (Watts, 1990; González-Sargas et al., 2024), poor soil quality after tailings removal, and residual elevated concentrations of Hg in Song Sparrow resulting from elevated Hg in invertebrate prey resources (Ackerman et al., 2024). Injury to Song Sparrow reproduction and survival was determined based on elevated Hg concentrations in egg tissue, with egg-equivalent Hg concentrations exceeding 1.8 µg Hg/g wet mass, resulting in severe injury to reproduction and survival (Ackerman et al., 2024).

Due to the effects of Hg on Song Sparrow reproduction and survival, the Trustees chose to use a REA (Table 1) based on the metric of discounted bird-years (DBYs). In REA, one bird lost or restored within a year is counted as a bird-year. When the relevant economic discount factor is applied (NOAA, 1999), the bird-years are converted to DBYs, which are then aggregated. In this example, the availability of local population data made it possible to measure changes in bird density from Hg contamination to determine an annual loss of DBYs per acre (called a density REA). REA can also be applied to a direct kill of animals and changes in life history (e.g., reduced hatching success, lower survival rates), which is referred to as a life history REA, as well as losses measured in weight, which is called a biomass REA. It was determined that an average of 95 DBYs per acre were lost across 250 acres, resulting in 23,750 total DBYs owed (i.e., 95 DBYs/acre × 250 acres). Given the established loss of DBYs (i.e., debit), riparian habitat restoration was proposed to increase on-site Song Sparrow density (i.e., credit). The Trustees were tasked with evaluating riparian habitat restoration options and associated costs, within the context of elevated background Hg concentrations, to estimate the expected gains in DBYs per acre over time. Although an oversimplification of typical NRDAR sites, on-site restoration needs to produce an average of 95 DBYs per acre to achieve the Trustees’ goals for return to baseline (debit = credit). To facilitate consideration of multiple restoration actions, Trustees treated the average uplift of 95 DBYs per acre as a “target” for restoration.

**Fig. 2:**
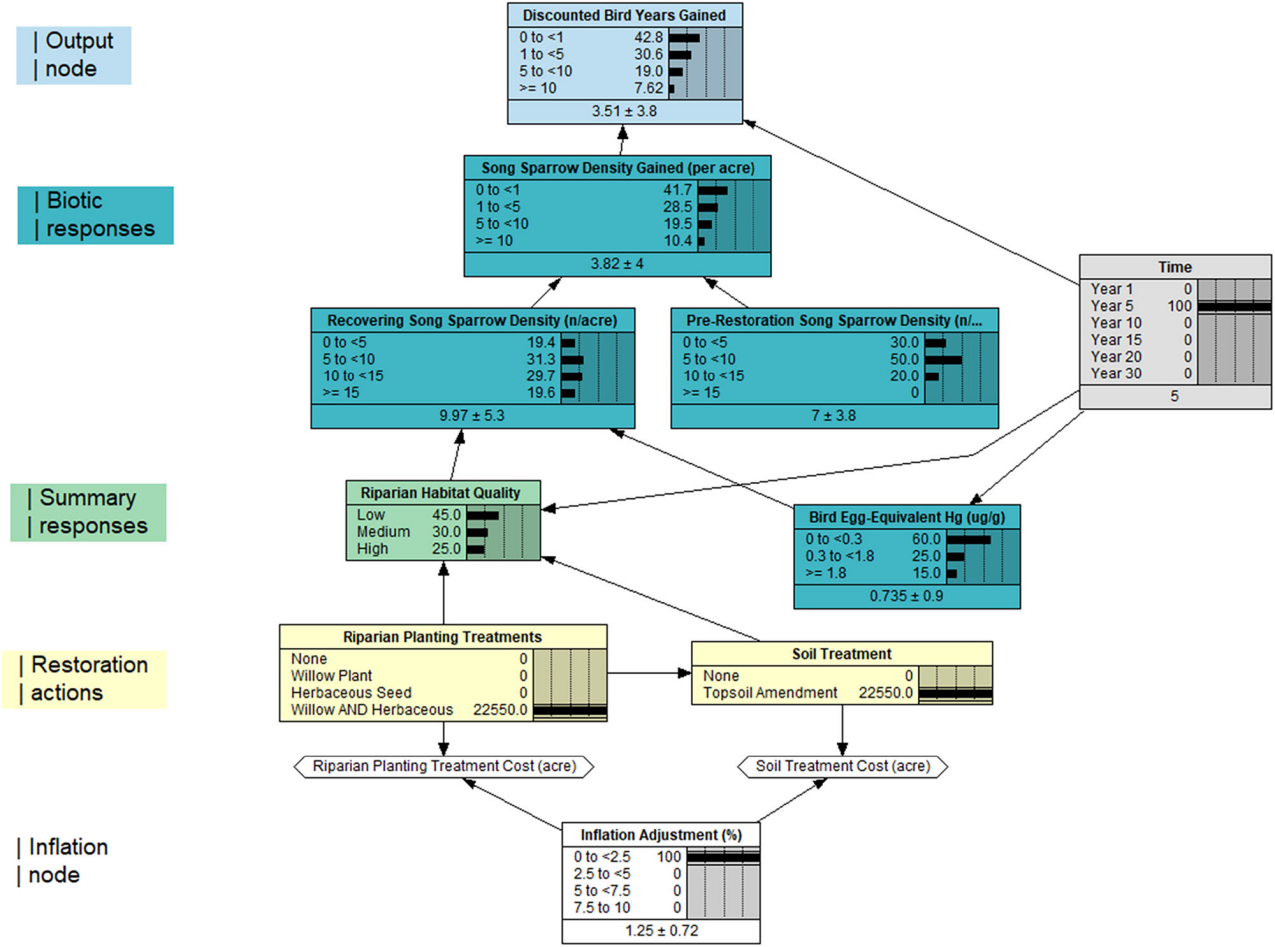
Bayesian decision network (BDN) illustrating the discounted bird years for Song Sparrow (*Melospiza melodia*) gained with riparian plantings and soil treatment restoration practices. The Song Sparrow model consisted of 12 nodes (variables) that included 2 decision nodes (with 6 decision conditions), 2 utility nodes, 15 links, and 372 conditional probability values. See Table S1a–c for details on node parameterizations. The inflation, summary responses, biotic responses, and output nature nodes show the likelihood (right side of a node) of the different node states (left side of a node). The restoration actions are decision nodes, which are selected in combination and indicate the cost of restoration given the assigned utility costs and inflation adjustment

We constructed a BDN to evaluate riparian habitat restoration actions targeted at increasing Song Sparrow densities (Fig. 2). Given degraded soil conditions and riparian plant communities, the restoration options considered soil amendments (“none” or “topsoil amendment”) and four planting options (“none,” “willow planting,” “herbaceous seeding” of native plants, or both “willow planting and herbaceous seeding”). These restoration actions were used in two separate decision nodes, “Riparian Planting Treatments” and “Soil Treatment.” Utility nodes were linked to the restoration decision nodes to incorporate the cost of the restoration treatment and an inflation adjustment (ranging from 0 to 10%). We linked restoration treatment decision nodes to a summary node that integrated riparian habitat quality for Song Sparrows as a node (scored as “low,” “medium,” and “high”) parameterized based on the different habitat restoration actions and projected changes in habitat quality over time (Fig. S1a). The habitat quality metric was defined by expected improvements in vegetation density, the type of vegetation established (willow vs. herbaceous, native vs. non-native), and a literature review of the relationship between riparian plants and Song Sparrow density and nesting success (Gardali et al., 1999; Shanahan et al., 2011; Rockwell and Stephens, 2018; Stephens and Rockwell, 2019; Campos et al., 2020; González-Sargas et al., 2024) (Table S1a–c). In addition, we assumed that soil amendment would enhance the growth of native herbaceous plants and that the no-restoration treatment (i.e., natural recovery) would have limited natural recovery. Time, represented from years 1 through 30, began post-remediation and ended immediately after restoration. Selecting “None” as the restoration decision aligns with the Trustees’ consideration of “natural recovery” to baseline, if possible.

We parameterized a bird egg-equivalent Hg node that bracketed reference toxicity values from Ackerman et al. (2024), which ranged from no injury (0 µg Hg/g wet mass) to severe injury (1.8 µg Hg/g wet mass) for bird survival and reproduction (Table S1a). We projected reduced egg-Hg concentrations from years 1 through 30 based on reduced Hg contamination following remedial activities (Fig. S1b). Using the joint probability of riparian habitat quality and bird egg Hg, we projected recovering Song Sparrow densities relative to riparian habitat quality and the effects of Hg bioaccumulation on survival and reproduction (Ackerman et al., 2024). In our stylized example, we provided a probability distribution for the pre-restoration Song Sparrow density (birds per acre), which we compared to the recovery of the Song Sparrow population to estimate the increase in bird density. We converted Song Sparrow density to DBYs by multiplying the number of Song Sparrows gained per acre by a discount factor (Table 1) calculated as (1/(1 + *r*)^time^), where *r* is the discount rate and time is relative to the base year. We assigned a discount rate of 3% and set the base year as year 0.

### Assessing Restoration Effectiveness: A Case Study of Habitat Restoration in the Upper Arkansas River, Colorado

The upper Arkansas River (UAR) watershed near Leadville, Colorado, was mined for heavy metals through the late 19th and early 20th centuries, which resulted in significant environmental degradation, including acid mine drainage, large deposits of mine tailings, fluvial tailings within the UAR floodplain, and substantial physical disturbance of the streambed and riparian areas. Remediation of acid mine drainage sources and fluvial tailings was started in 1983 and was completed by 2008. Significant improvements in benthic macroinvertebrate communities and the brown trout (*S. trutta*) fishery were observed in response to improved water quality (Clements et al., 2010). Injury to brown trout within an 18-km reach of the UAR was established based on brown trout fry mortality, brown trout population reduction, reduction in mayfly abundance, and injury to brown trout through behavioral avoidance of metal-polluted river habitats. Lost ecological services were calculated, and a habitat equivalency analysis was used to estimate the discounted service acre-years (DSAYs) owed. Following the NRDAR settlements, instream habitat restoration, bank stabilization, and riparian vegetation were completed by 2014. Researchers have since evaluated changes in instream habitat (Richer et al., 2019), riparian vegetation (Cubley et al., 2022), brown trout (Richer et al., 2022), benthic macroinvertebrates (Wolff et al., 2022), and food web bioenergetics (Kotalik et al., 2023).

We constructed a BDN that evaluated changes in stream habitat, as well as the associated responses by benthic macroinvertebrates and brown trout, before and after restoration in an 8-km section of the 18-km UAR NRDAR reach. The decision nodes in the model were used to implement restoration (instream habitat restoration and bank stabilization) or not implement restoration (remediation only). Based on changes in stream habitat reported by Richer et al. (2019) before (2013) and after (2014–2016) restoration, we assigned probability distributions for the weighted usable area (WUA) for trout fry, juveniles, and adults (m^2^/meter), average habitat heterogeneity (i.e., the coefficient of variation for stream depth and velocity), and abundance of brown trout foraging positions (n/m) (Table S1d, e). We assumed that changes in habitat metrics associated with instream habitat and bank stabilization were approximately equivalent when implemented independently and additive when implemented together (Roni et al., 2008). We also assumed that bank stabilization was not associated with foraging position and that changes in stream habitat post-restoration were constant. We related the implementation of instream habitat restoration to all three habitat metrics and bank stabilization to changes in WUA and average habitat heterogeneity. The three habitat response nodes were merged into an integrated stream habitat quality metric using literature and expert knowledge to estimate probabilities of stream habitat quality (Table S1d, e). We assumed changes in instream habitat in response to restoration implementation remained constant over time for this model but acknowledge that changes in physical habitat often occur after restoration completion (e.g., stream channel migration, shifting substrate composition).

We parameterized the benthic macroinvertebrate biomass node based on observations from Kotalik et al. (2023), which evaluated the responses of benthic macroinvertebrates to habitat restoration in the UAR. We assumed relatively quick responses of benthic macroinvertebrates to restoration, given their short life cycle, ability to rapidly recolonize, and previous observations of benthic communities following restoration in the UAR (Wolff et al., 2022; Kotalik et al., 2023). The outcome node, brown trout biomass (based on trout greater than 1 year of age), was parameterized based on the joint probability of benthic macroinvertebrate biomass (prey availability), stream habitat quality, and time. Time at year 0 represented restoration completion, year 0 to 5 represented post-restoration evaluation of effectiveness based on field monitoring (Richer et al., 2022; Kotalik et al., 2023), and year 6 to 30 represented projected changes in brown trout biomass. Based on the assumption that habitat quality and benthic biomass remained constant after restoration, we projected changes in trout biomass based on growth trajectories, carrying capacity reported in the literature (Bowlby and Roff, 1986; Kwak and Waters, 1997), and trout biomass observed at other sites in the UAR (Richer et al., 2022). BDNs incorporated uncertainty through the prior likelihoods assigned to each node and the conditional probability tables that were incorporated into the output.

### Model Structure

**Fig. 3:**
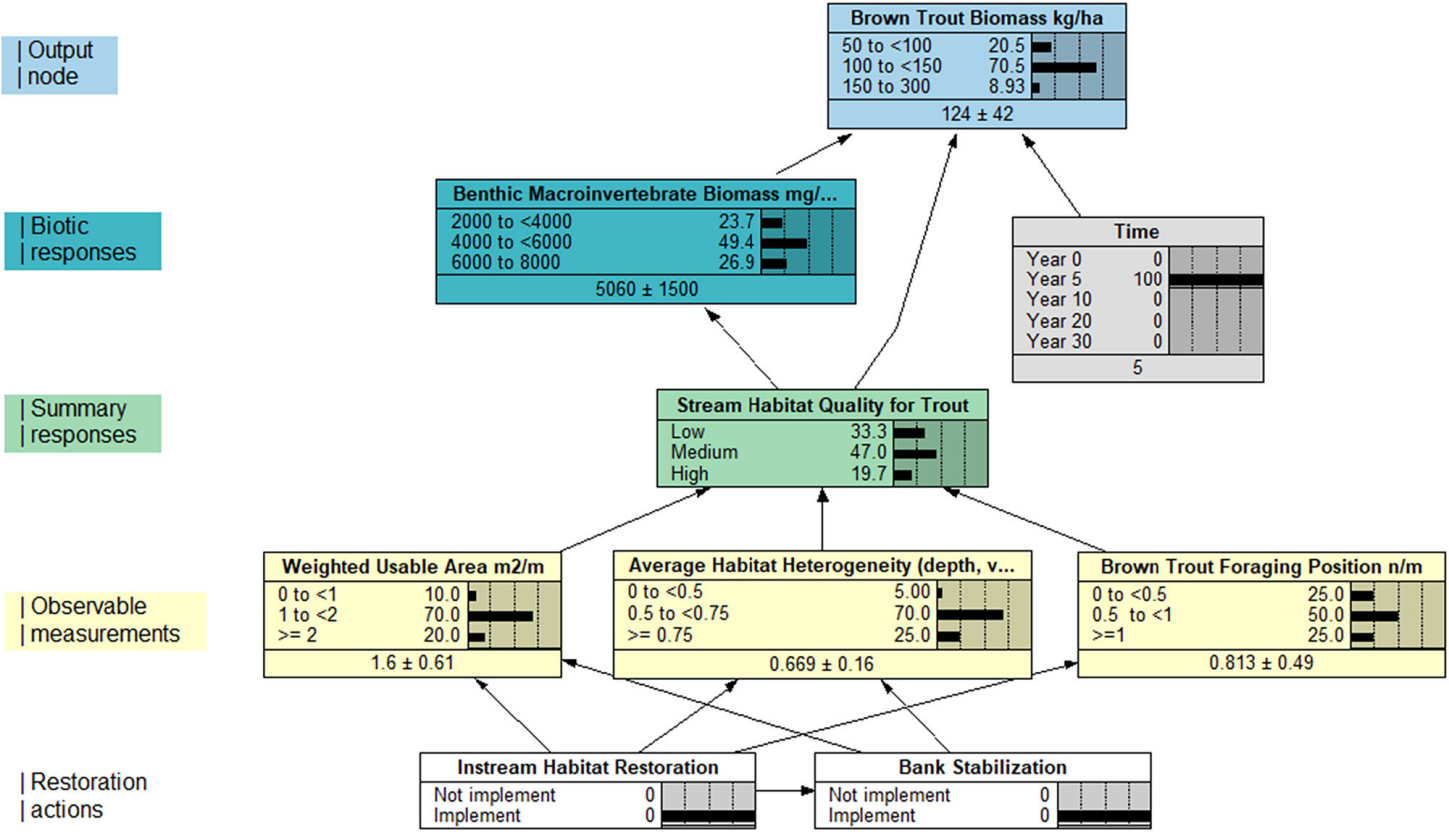
Bayesian decision network showing Brown Trout (*Salmo trutta)* biomass gained with instream habitat restoration and bank stabilization. The Brown Trout model consisted of 9 nodes that include 2 decision nodes (with 4 decision conditions), no utility nodes, 13 links, and 260 conditional probability values. See Table S1d, e for details on node parameterizations. The observable measurements, summary responses, biotic responses, and output nodes show the likelihood (right side of a node) of the different node states (left side of a node). The restoration actions are decision nodes, which are selected in combination

Our two BDN models differed in structure and objectives. The Song Sparrow model was designed to illustrate pre-settlement restoration planning, and the UAR model was developed to assess and forecast the effectiveness of restoration. The Song Sparrow model consisted of 12 nodes (variables) that included 2 decision nodes (with 6 decision conditions), 2 utility nodes, 15 links, and 372 conditional probability values (Figs. 2 and SI-1). Two biotic response outcome nodes provide probabilities of 4 state-ranges of Song Sparrow density gained (n/acre) and 4 state-ranges of DBYs gained. The Brown Trout model consisted of 9 nodes that include 2 decision nodes (with 4 decision conditions), no utility nodes, 13 links, and 260 conditional probability values (Figs. 3 and SI-1). The biotic response outcome node provided probabilities of 3 state-ranges of brown trout biomass (kg/stream ha). Both models also included a time variable that projected biotic responses at years 0 or 1, 5, 10, 20, and 30.

## Results

### Song Sparrow BDN

**Fig. 4:**
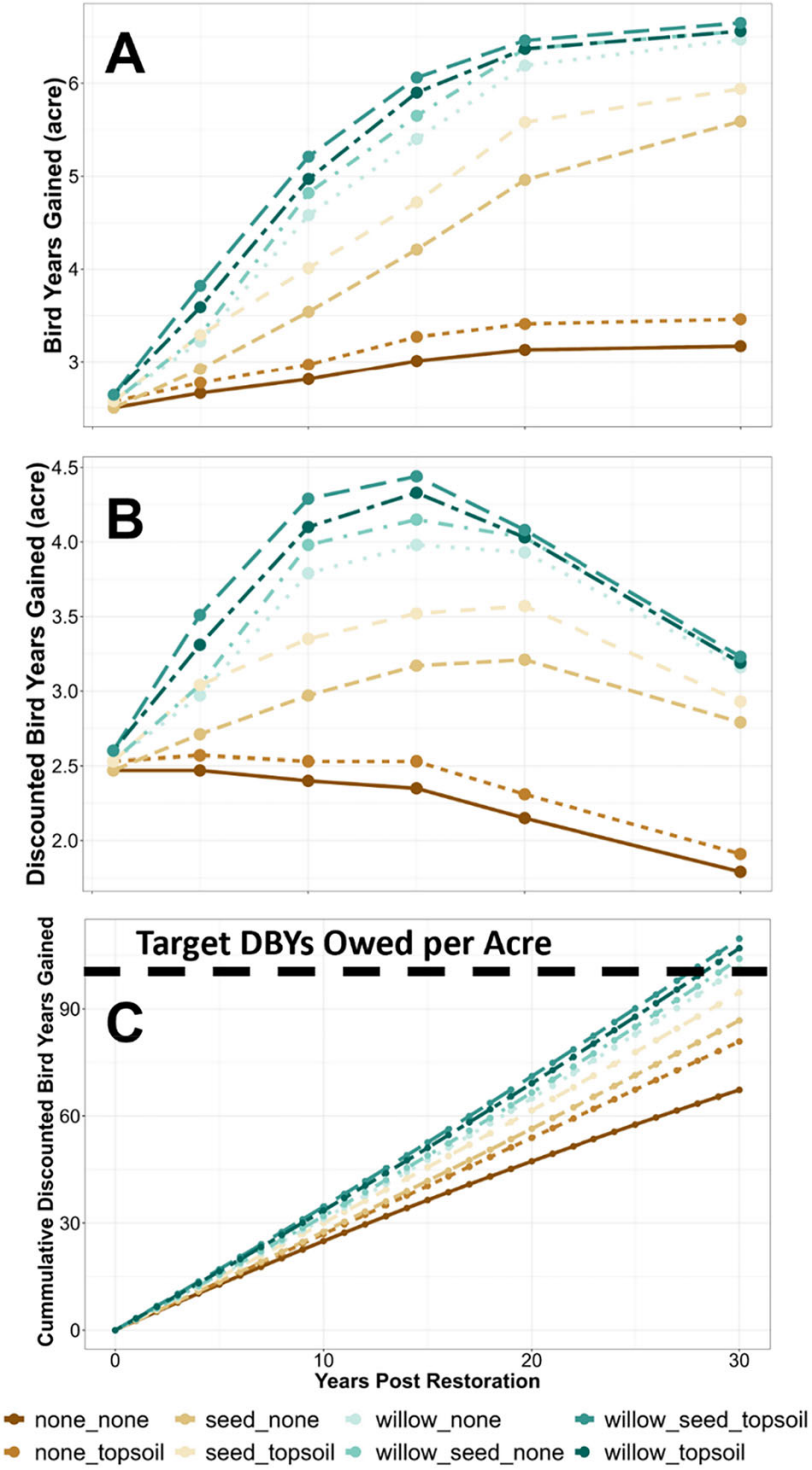
Model-predicted Song Sparrow (*Melospiza melodia*) bird-years gained (**A**), discounted bird-years (DBYs) gained (**B**), and cumulative DBYs gained among restoration treatments (**C**). Lines track the model-predicted data points over time

The Song Sparrow BDN included eight possible decisions, ranging from no restoration to combined willow planting, herbaceous seeding, and topsoil amendment. The expected values in bird years gained varied considerably across the eight different restoration scenarios (Fig. 4a), with minimal gains through natural recovery (i.e., no plantings and no amendments). In contrast, relatively greater increases in DBYs were observed in all cases where willow plantings were included in restoration, regardless of soil treatment. Variation in DBYs gained among restoration treatments increased up until year 15, where the greatest variation was observed, and then decreased through the end of the projected assessment period (year 30) (Fig. 4b). The inflection points at which decreases in annual DBYs gained were observed over time varied among restoration treatments, with decreases in DBYS occurring at year 10 for no restoration and topsoil only, compared to year 15–20 for all other treatment combinations.

**Fig. 5:**
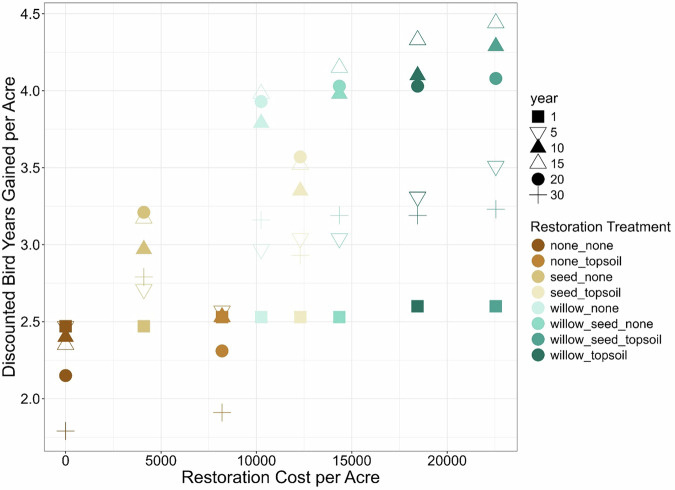
Discounted Bird-Years (DBYs) gained relative to restoration costs ($ USD), restoration treatment (colors), and year of post-restoration (symbols)

**Fig. 6:**
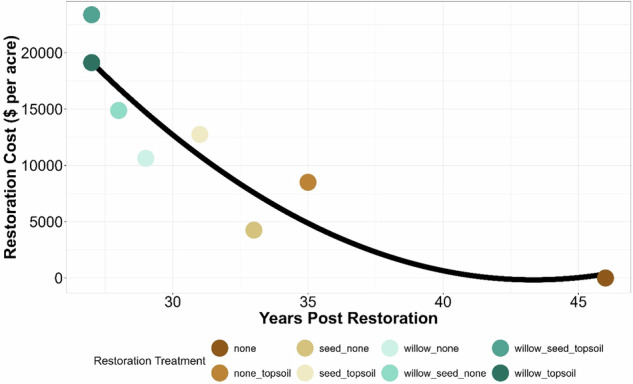
Years required to reach baseline (Discounted Bird Years Gained; *n* = 95 per acre) among the different restoration treatments relative to restoration cost ($ USD) per acre

Cumulative DBYs gained over time showed the target of 95 DBYs gained per acre was met and exceeded for 5 of the 8 restoration actions by year 30 (Fig. 4C). With natural recovery, it was projected to take 46 years to reach 95 DBYS per acre, whereas the combined willow planting, herbaceous seeding, and topsoil amendment treatment took ~26 years. Based on the time required with natural recovery to reach baseline (46 years), nearly double the number of DBYs would be gained with the top restoration treatment. These results suggest that all the restoration actions could restore the system beyond baseline over time (credit > debit). Generally, as the cost of the different restoration treatments increased, the DBYs increased (Fig. 5), and the years required to reach baseline decreased (Fig. 6). Thus, decision-makers can consider the trade-offs of a lower-cost restoration action across most of the 250 acres versus a more productive, but more expensive restoration on fewer acres, as well as potential combinations of restoration actions. The debit divided by credit per acre for these restoration actions resulted in the number of acres owed for the restoration (called scaling in NRDAR, where debit equals credit). This pre-settlement restoration planning provided an opportunity to review restoration scenarios during settlement negotiations.

### Brown Trout BDN

**Fig. 7:**
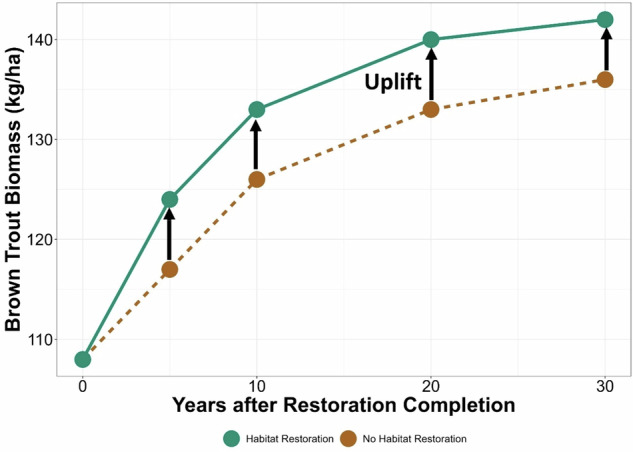
Modeled Brown Trout (*Salmo trutta*) biomass over time with and without restoration treatments. Lines track the modeled expected values in Brown Trout biomass. Black arrows indicate the uplift (positive increase in Brown Trout biomass) resulting from habitat restoration actions compared to a no-habitat restoration scenario in our Bayesian decision network output

In the Brown Trout model, implementation of instream habitat restoration and bank stabilization resulted in marginal increases in the modeled expected values of WUA from 1.3 (±0.49 standard deviation [s.d.]) to 1.6 (±0.62) and average habitat heterogeneity from 0.525 (±0.21) to 0.575 (±0.42). Foraging position by brown trout with implementation of instream habitat restoration increased from 0.575 (±0.42) to 0.813 (±0.49). The dominant state of the stream habitat quality node remained “medium” with and without restoration; however, the posterior distribution shifted the probability of “high” stream habitat quality from 1 to 20% with implementation of both restoration options (SI-2). The expected values of benthic macroinvertebrate biomass, which are the dominant prey consumed by Brown Trout in the UAR (Kotalik et al., 2023), increased by 6% from 4820 to 5060 mg/m^2^. With restoration, the expected biomass of Brown Trout increased from the time of restoration completion (year 0) to year 5, from 108 (±28) to 124 (±42) kg/ha (Fig. 7). Without restoration, increases in Brown Trout biomass were still expected, given the benefits of improved water quality in the UAR (Clements et al., 2010); however, the expected increase in biomass was lower, at 117 (±36) kg/ha in year 5. With the expected uplift from restoration, it took 10–15 fewer years to reach levels of Brown trout biomass compared to without restoration (Fig. 7).

## Discussion

### Broad Applicability of the BDN Framework

We used two case studies to demonstrate the value of BDNs in modeling NRDAR and to highlight key considerations for using BDN model forecasting to inform restoration planning and to evaluate restoration effectiveness. Bayesian Networks have been applied to various aspects of natural resource management and restoration. Our approach here expands such tools by explicitly including management decisions and utility costs in the BDN framework. The expected values of alternative restoration decisions are also shown to vary by decision, and the framework provides a straightforward way to explore tradeoffs of costs and outcomes among alternative decision pathways. Furthermore, the BDN approach highlights the implications of uncertainty in environmental responses and the propagation of all evidence and uncertainties throughout the system (Barrientos and Vargas, 1998; Borsuk et al., 2004), thereby enabling the identification of key knowledge gaps that have high uncertainty (Holzkämper et al., 2012). Below, we outline best practices for developing BDNs for restoration planning and evaluating restoration effectiveness, including a discussion of the advantages and cautions of the BDN approach for NRDAR decision-making and restoration decision-making. We also discuss how BDNs can be helpful in value of information (VOI) analyzes.

### Restoration Planning and Forecasting

Our application of BDNs to restore injured Song Sparrow densities highlighted the power and flexibility of using decision advisory networks to plan and design restoration actions, ensuring that implementation can meet the goals of NRDAR practitioners and other natural resource managers. In terms of ecological complexity, the approach enabled us to incorporate key functional relationships pertinent to restoration outcomes of the injured natural resource. For example, we incorporated mercury exposure alongside riparian habitat quality in evaluating the recovery of the Song Sparrow population density. For contaminated sites with remedial activity either before or concurrently with restoration, the ability to model the interactive influence of residual contaminant exposure when planning and forecasting restoration responses is a significant benefit of the causal network approach. BDNs are flexible enough to incorporate multiple contaminant exposure nodes (e.g., trace metals, persistent organic pollutants), enabling practitioners to address differences in projected exposure scenarios.

The BDN we developed incorporated economic information that allowed for assessment of restoration uplift given the varying costs of restoration actions, resulting in more “trade-off” analyzes for decision making. For example, the most intensive restoration treatment (seeding, willow plantings, and topsoil amendments) greatly accelerated Song Sparrow recovery such that DBYs gained were ~2× higher over 30 years compared to the least intensive options (no action or topsoil amendment alone). The most intensive treatments also required approximately half the amount of time to reach baseline (26 years) compared to no action (46 years), but these intensive treatments also came with the highest restoration cost ($23,375/acre). Other restoration actions, such as willow planting alone, which cost approximately half ($10,625/acre), took only three more years to reach baseline. For NRDAR practitioners faced with making decisions on what restoration actions to take, BDNs allow for identifying an “optimal” combination of actions that can balance cost, model-predicted recovery (e.g., DBYs gained), and recovery time. Given the diversity of NRDAR cases, the “optimal” set of restoration actions will vary. For example, a practitioner may be faced with selecting restoration actions given a desired timeframe for recovery at the lowest cost, or conversely, the fastest recovery within a fixed budget. A major benefit of BDNs is that different combinations of restoration actions can be assessed relative to one another, allowing practitioners to evaluate different options to meet their specific restoration goals.

### Evaluation of Restoration Effectiveness and Forecasting

Once habitat restoration is implemented, restoration practitioners need to evaluate the effectiveness while also projecting future expected restoration outcomes. The UAR is unique in that there have been robust evaluations of restoration effectiveness on habitat, aquatic invertebrates, and Brown Trout (Richer et al., 2019; Wolff et al., 2022; Richer et al., 2022; Kotalik et al., 2023); however, the responses of these metrics have not been evaluated using a modeling approach that explicitly linked all these variables together. Using a BDN approach, we concurrently evaluated the direct and indirect effects of habitat restoration on prey resources (i.e., benthic macroinvertebrates) and Brown Trout using empirical relationships associated with pre- and post-restoration monitoring. This approach allowed us to mechanistically link changes in habitat with biological responses. We simultaneously used the underlying model structure and parameterization, along with observed responses to restoration in the first 5 years, to inform projections of Brown Trout biomass uplift over a 30-year time frame.

As additional monitoring data are collected on the UAR, the BDN node parameterization and projections can be updated to reflect how the ecosystem is changing and is projected to change. For example, we assumed that changes in instream habitat after restoration were constant in the UAR BDN; however, rivers are dynamic systems, and riverine habitats change naturally over time (Wohl et al., 2005). The metrics for instream habitat in the UAR BDN can be updated with future evaluations, or if restoration practitioners are interested in evaluating how changes in habitat might influence aquatic life, “influence run” analyzes where specified nodes are set to their extreme state conditions, and the probability results of a specified output node in the model are recorded, can help inform potential changes (SI-2*, “UAR influence run analysis”*). In addition, projections of brown trout and benthic biomass for years 10–30 were based on the observed responses from years 0–5. Future monitoring can be used to update and inform recovery trajectories and the carrying capacity of the UAR. The ability to comprehensively evaluate, update, and project future changes provides practitioners with a valuable tool to inform present and expected future restoration success. Where relevant, the outputs from the evaluation of restoration effectiveness at this site could be used to inform similar restoration projections at other sites. On the other hand, projections of habitat changes can be tailored to specific restoration cases, taking into account site-specific characteristics, data availability, and expected recovery trajectories.

### Best Practices for Building BDNs for Restoration Planning and Evaluation of Restoration Effectiveness

The following are several guidelines to help ensure that causal network models developed for NRDAR and other restoration and remediation applications are effective as decision-advisory tools. First, expert knowledge and experience should be used in creating the causal network structure, including definitions of nodes (variables) and their states. Our model construction involved iterative feedback from NRDAR practitioners (scientists and economists), modelers, and ecotoxicologists, as well as an extensive literature review. Second, empirical data, as available (e.g., derived from in situ studies or monitoring), should be used to set model parameter values for state range values and categories, as well as values of conditional probability tables. Where empirical data are unavailable, then the use of expert knowledge through structured elicitation procedures (Hanea et al., 2021; Marcot, 2019) would be used because the structure and parameters of BNs and BDNs can be based on a combination of empirical data and expert knowledge (Constantinou et al., 2016). Next, the inclusion of decision and utility nodes, as illustrated in our two model examples, can aid in evaluations of restoration costs, both pre- and post-settlement. A functional BDN advisory model can then be assessed for parameter sensitivity to help identify nodes that may be contributing most to the outcomes but may be least well understood (Peng et al., 2022). That is, the parameters that have the greatest uncertainty and spread of their probability values, as well as the influence of extreme states on the output node, can be identified (see SI-2; “Comparison of Song Sparrow Model and Brown Trout Model for model outcome sensitivity and parameter influence”). This additional information may help NRDAR practitioners and other resource managers prioritize variables and conditions that require further study, survey, and/or monitoring, to inform observations, which are then fed back into the model to update the structure and/or parameters (Marcot, 2012). Finally, BN/BDN experts are needed to help avoid some common pitfalls in network model development, such as dealing with latent variables, outlier expert judgments, model peer review, model calibration and validation, model overfitting, and other concerns (Marcot, 2017). In general, applying a precautionary principle (Varis and Kuikka, 1997) can help ensure that the decision to invest in the development of probability models such as BNs/BDNs will return information for practitioners to successfully address the challenges encountered in restoration planning, as well as evaluation of restoration effectiveness.

### Use of BDNs in Estimating the Value of Information

Information is the foundation of evidence-based decision-making and action. Producing and sharing information with decision-makers on the restoration life cycle has value, in part, because it facilitates an understanding of the costs and benefits of investments. Through several case studies, the U.S. Geological Survey has demonstrated that a BDN approach is particularly useful for assessing changes in outcomes and associated benefits in response to information (Pindilli et al., 2023; Pindilli and Loftin, 2022; Pearlman et al., 2019). Decision pathways and assumptions were identified for actions taken “with” and “without” the information. Decision scenarios with next-best information (“without” the information products being evaluated) are typically referred to as counterfactuals. The measurement of the effect of the additional information relative to a counterfactual is an estimate of the societal benefits from the reduced uncertainty due to the information.

The BDN modeling approach provides an explicit means of developing a VOI analysis based on variation in restoration actions. The Song Sparrow model illustrates how the utility cost nodes for riparian planting and soil treatment options can provide a means for adjusting the outcomes and costs of restoration actions for VOI analysis. The results display the probabilities and resulting expected values for given management decisions and the biotic outcome, including Song Sparrow density and the number of DBYs gained. The modeling approach also provides a framework in which various sources of information can be used to structure and parameterize the network, including empirical data, expert knowledge, and literature. Our models used all three of these sources. The modeling approach provides flexibility in representing diverse contaminant scenarios, different restoration management actions, and expected outcomes to develop both “with” and “without” scenarios. Conceptually, the structure and outputs of BDNs could be incorporated into VOI analyzes to determine the economic benefits/costs of choosing one restoration action over another (including no action), the avoided losses in resources through remediation and restoration, and the expected benefits of additional study to inform investment in gathering more data and information.

## Conclusion

The application of causal network modeling captured the key elements of restoration planning and evaluation of restoration effectiveness, as illustrated through two NRDAR case studies. The approach facilitated productive, multidisciplinary team discussions, literature reviews, identification and use of available empirical data, and the incorporation of expert knowledge and experience to identify model components and their causal relationships. Contaminant-impacted sites are complex, varying in stressors and contaminants, species and habitat types, and restoration actions planned and taken. While the BDNs we developed were specific to two case studies involving Song Sparrow and Brown Trout, the structure is adaptable to a diversity of sites, natural resources, and actions. Importantly, constructing and interpreting BDNs is a complex process that requires time, resources, and specialized expertise, which is an important consideration when using BDNs alongside other methods of risk assessment and decision-making. Nevertheless, we suggest that causal network modeling can provide practitioners with a decision-advisory tool useful for a wide range of projects focused on mitigation, reclamation, remediation, or restoration.

## Supplementary information

Supplementary Information

## Data Availability

All Bayesian Network models will be archived in the Bayesian Network Modelling Association repository upon acceptance.
