# Correction: Causal Networks to Inform Decisions for Ecological Restoration

**DOI:** 10.1007/s00267-026-02411-6

**Published:** 2026-03-21

**Authors:** Christopher J. Kotalik, Freya E. Rowland, Bruce G. Marcot, Kristin E. Skrabis, David M. Walters, Jo Ellen Hinck, William H. Clements, Eric E. Richer, John P. Isanhart

**Affiliations:** 1 U.S. Geological Survey, Columbia Environmental Research Center, Columbia, MO USA; 2 U.S. Forest Service, Pacific Northwest Research Station, Portland, OR USA; 3 U.S. Department of the Interior, Office of Policy Analysis, Washington, DC USA; 4 U.S. Geological Survey, Natural Hazards Mission Area, Reston, VA USA; 5 Department of Fish, Wildlife, and Conservation Biology, Colorado State University, Fort Collins, CO USA; 6 Aquatic Research Section, Colorado Parks and Wildlife, Fort Collins, CO USA; 7 U.S. Department of the Interior, Office of Restoration and Damage Assessment, Denver, CO USA

Correction to: Environmental Management 10.1007/s00267-025-02323-x

The article “Causal Networks to Inform Decisions for Ecological Restoration”, written by Christopher J. Kotalik, Freya E. Rowland, Bruce G. Marcot, Kristin E. Skrabis, David M. Walters, Jo Ellen Hinck, William H. Clements, Eric E. Richer and John P. Isanhart was originally published under the copyright “This is a U.S. Government work and not under copyright protection in the US; foreign copyright protection may apply 2025”. As a result of the subsequent decision to publish the article under the open access model, the article’s copyright notice was changed on 6 January 2026 to © William H. Clements, Eric E. Richer. Parts of this work were authored by US Federal Government authors and are not under copyright protection in the US; foreign copyright protection may apply and the article is now distributed under a Creative Commons Attribution-NonCommercial-NoDerivatives 4.0 International License, which permits any non-commercial use, sharing, distribution and reproduction in any medium or format, as long as you give appropriate credit to the original author(s) and the source, provide a link to the Creative Commons licence, and indicate if you modified the licensed material. You do not have permission under this licence to share adapted material derived from this article or parts of it. The images or other third party material in this article are included in the article’s Creative Commons licence, unless indicated otherwise in a credit line to the material. If material is not included in the article’s Creative Commons license and your intended use is not permitted by statutory regulation or exceeds the permitted use, you will need to obtain permission directly from the copyright holder. To view a copy of this license, visit http://creativecommons.org/licenses/by-nc-nd/4.0/.

