# Supplementary material for: Causal Networks to Inform Decisions for Ecological Restoration
Source: Environ Manage. 2025 Nov 20;76(1):7. doi: 10.1007/s00267-025-02323-x (PMC12995969; doi:10.1007/s00267-025-02323-x)
Supplement: Supplementary file 1 — Supplementary Information [file 267_2025_2323_MOESM1_ESM.docx]

**Supplementary Information**

**Causal networks to inform decisions for ecological restoration**

Christopher J. Kotalik,^1*#^ Freya E. Rowland,^1#^ Bruce G. Marcot,^2^ Kristin E. Skrabis,^3^ David M. Walters,^1^ Jo Ellen Hinck,^4^ William H. Clements,^5^ Eric E. Richer,^6^ John P. Isanhart^7^

^1^U.S. Geological Survey, Columbia Environmental Research Center, Columbia, MO, USA

^2^U.S. Forest Service, Pacific Northwest Research Station, Portland, OR, USA

^3^U.S. Department of the Interior, Office of Policy Analysis, Washington, DC, USA

^4^U.S. Geological Survey, Natural Hazards Mission Area, Reston, VA, USA

^5^Department of Fish, Wildlife, and Conservation Biology, Colorado State University, Fort Collins, CO

^6^Aquatic Research Section, Colorado Parks and Wildlife, Fort Collins, CO

^7^U.S. Department of the Interior, Office of Restoration and Damage Assessment, Denver, CO, USA

*Corresponding Author: [ckotalik@usgs.gov](mailto:ckotalik@usgs.gov)

#: co-first authors

**SI-S1**

**Table S1a-c:** Song Sparrow BDN model documentation

**Figure S1a-b:** Song Sparrow BDN node summaries and model output

**Table S1d-e:** Brown Trout BDN model documentation

**SI-S2**
Sensitivity analysis and influence run analysis for Song Sparrow and Brown Trout BDNs

“Any use of trade, firm, or product names is for descriptive purposes only and does not imply endorsement by the U.S. Government."

**SI-S1**

**Table S1a.** Parameters, parameter states, justification for inclusion, and references to support the development of the Song Sparrow (*Melospiza melodia*) Bayesian decision network.

| **Model Parameter** | **Parameter States** | **Justification for inclusion** | **References** |
| --- | --- | --- | --- |
| **Biotic responses** | | | |
| **Song Sparrow Density Gained (per acre)** | 0 to < 1 | Composite variable of gains in Song Sparrow density over time. Populated by estimating the probability of difference between pre-restoration and recovering Song Sparrow density nodes. | Not applicable |
|  | 1 to < 5 |  |  |
|  | 5 to < 10 |  |  |
|  | ≥ 10 |  |  |
| **Recovering Song Sparrow Density (n/acre)** | 0 to < 5 | Song Sparrows are quick to respond to restoration efforts. We reviewed the literature and found density estimates for Song Sparrows in arid or semi-arid streams in the western United States or Baja California to estimate recovering population densities that are typically observed. Post-restoration measurements of Song Sparrow densities in the literature are near 18-20 birds/acre. | Gonzalez-Sargas et al. 2024; Dybala et al. 2018; Campos et al. 2020 |
|  | 5 to < 10 |  |  |
|  | 10 to < 15 |  |  |
|  | ≥ 15 |  |  |
| **Pre-Restoration Song Sparrow Density (n/acre)** | 0 to < 5 | Values and distribution of Song Sparrow adults per acre from a hypothetical site; Song Sparrow densities are based on reported pre- and post-restoration density from arid or semi-arid montane riparian meadows in California (Campos et al. 2020) and the Central Valley, California (Dybala et al. 2018). We chose to model adults because nest success can be highly variable (Stephens and Rockwell, 2019), and adults represent significant contributions to the population. A literature review showed that pre-restoration Song Sparrow densities averaged around 5 birds/acre. | Gonzalez-Sargas et al. 2024; Dybala et al. 2018; Campos et al. 2020; Stephens and Rockwell 2019 |
|  | 5 to < 10 |  |  |
|  | 10 to < 15 |  |  |
|  | ≥ 15 |  |  |
| **Bird Egg-Equivalent Hg (µg/g)** | 0 to < 0.3 | Changes in Song Sparrow egg Hg concentrations (µg/g fresh wet weight (fww)) bracket projected reductions associated with remedial actions taken place on-site before restoration; Song Sparrow egg Hg concentrations reference Ackerman et al. 2024 for no injury (0 ug/g fww)), moderate injury (EC5) (0.30 ug/g fww), and severe injury (EC20) (1.8 ug/g fww) for combined survival and reproduction, respectively. We assumed Hg concentration in Song Sparrow would be elevated immediately after remediation completion and the start of restoration assessment period, but Hg concentration in Song Sparrows would decline over time. This assumption was based on previous research that has shown residual Hg takes time to cycle through terrestrial food webs after remediation, due in part to inorganic Hg being mobilized during restoration construction and the conditions created within the restoration that promote Hg methylation. | Eckley et al. 2020; Ackerman et al. 2024 |
|  | 0.3 to < 1.8 |  |  |
|  | ≥ 1.8 |  |  |
| **Summary responses** | | | |
| **Riparian Habitat Quality for Song Sparrows** | Low | An integrated stream habitat metric that estimates the habitat quality for Song Sparrows based on soil amendment (none vs. topsoil); planting options (none, herbaceous seeding, willow planting, and herbaceous AND willow planting); and time since restoration. We assumed that more time would lead to greater plant growth. | Rockwell and Stephens 2018; Gonzales-Sargas et al. 2024; Shanahan et al. 2011; Gardali et al. 1998; Watts 1991; Germain et al. 2018 |
|  | Medium |  |  |
|  | High |  |  |
| **Utility nodes** | | | |
| **Riparian Planting Treatment Cost (acre)** | Not applicable | This utility node assigns a value to the model based on a combination of the costs associated with no restoration, willow planting, herbaceous seeding, or both willow and herbaceous seeding restoration options (decision node), with an inflation adjustment (nature node). | Contract and bid estimates from the Upper Arkansas River, Colorado, Natural Resource Damage Aquatic Habitat Enhancement and Colorado River, Colorado, Habitat Restoration (CPW); Zentner et al. 2003 |
| **Soil Treatment Cost (acre)** | Not applicable | This utility node assigns a value to the model based on a combination of the costs associated with no soil amendment or topsoil amendment at the time of planting, as restoration options (decision node), with an inflation adjustment (nature node). | Contract and bid estimates from the Upper Arkansas River, Colorado, Natural Resource Damage Aquatic Habitat Enhancement and Colorado River, Colorado, Habitat Restoration (CPW); Zentner et al. 2003 |
| **Time variable** | | | |
| **Time** | Year 1 | Year 1 represents the onset of restoration. We assumed all herbaceous seeding and willow plantings were done this year. Time is used to describe changes in Hg concentrations in Song Sparrow eggs, riparian habitat quality for Song Sparrows, and the discounting of bird years gained over the restoration period. Discounting is an economic valuation where present and past resources are valued more highly than those in the future. | Julius 1999; Horsch et al. 2023; Baker et al. 2020; Eckley et al. 2020; |
|  | Year 5 |  |  |
|  | Year 10 |  |  |
|  | Year 15 |  |  |
|  | Year 20 |  |  |
|  | Year 30 |  |  |
| **Inflation adjustment (%)** | | | |
| **Riparian restoration cost inflation adjustment** | 0 to < 2.5 | Price adjustment to account for the increasing prices of restoration, bracketing annual inflation increases. |  |
|  | 2.5 to < 5 |  |  |
|  | 5 to < 7.5 |  |  |
|  | 7.5 to 10 |  |  |
| **Restoration Decision Nodes** | | | |
| **Riparian Planting Treatments** | None | Song Sparrows are highly dependent upon riparian vegetation for nesting and protection from predation, have a positive density response to more dense vegetation, and respond positively to the diameter at breast height of shrubs and trees in their habitat.  Willow planting is defined as the planting of 30-inch or taller rooted saplings in the riparian area. Herbaceous seeding is defined as seeding open soil with herbaceous plant seeds native to the region in riparian areas. | Rockwell and Stephens 2018; Gonzales-Sargas et al. 2024; Shanahan et al. 2011; Gardali et al. 1998; Watts 1991 |
|  | Willow Planting |  |  |
|  | Herbaceous Seeding |  |  |
|  | Willow AND Herbaceous |  |  |
| **Soil Treatment** | None | Degraded soil condition after the removal of contaminated sediments and mine tailings can be restored via topsoil amendment. The options are “None” for not adding any amendment or “Topsoil Amendment” for topsoil additions at the time of planting. | Zentner et al. 2003 |
|  | Topsoil Amendment |  |  |

**Table S1b.** Conditional probability tables for all nodes in the bird mercury decision network to assess restoration alternatives and increase Song Sparrow density. The posterior probability values, shown on a scale of [0,1], of the response node are listed in the first variable column set, and the respective state values of the parent nodes are listed in columns to the right of those.

| **Discounted Bird Years Gained** | | | | | |
| --- | --- | --- | --- | --- | --- |
| **0 to < 1** | **1 to < 5** | **5 to < 10** | **≥ 10** | **SongSparrowsGained** | **Time** |
| 1 | 0 | 0 | 0 | 0 to < 1 | Year 1 |
| 1 | 0 | 0 | 0 | 0 to < 1 | Year 5 |
| 1 | 0 | 0 | 0 | 0 to < 1 | Year 10 |
| 1 | 0 | 0 | 0 | 0 to < 1 | Year 15 |
| 1 | 0 | 0 | 0 | 0 to < 1 | Year 20 |
| 1 | 0 | 0 | 0 | 0 to < 1 | Year 30 |
| 0.011 | 0.989 | 0 | 0 | 1 to < 5 | Year 1 |
| 0.039 | 0.961 | 0 | 0 | 1 to < 5 | Year 5 |
| 0.078 | 0.922 | 0 | 0 | 1 to < 5 | Year 10 |
| 0.128 | 0.872 | 0 | 0 | 1 to < 5 | Year 15 |
| 0.232 | 0.768 | 0 | 0 | 1 to < 5 | Year 20 |
| 0.342 | 0.658 | 0 | 0 | 1 to < 5 | Year 30 |
| 0 | 0.032 | 0.968 | 0 | 5 to < 10 | Year 1 |
| 0 | 0.166 | 0.834 | 0 | 5 to < 10 | Year 5 |
| 0 | 0.346 | 0.654 | 0 | 5 to < 10 | Year 10 |
| 0 | 0.558 | 0.442 | 0 | 5 to < 10 | Year 15 |
| 0 | 0.799 | 0.201 | 0 | 5 to < 10 | Year 20 |
| 0 | 1 | 0 | 0 | 5 to < 10 | Year 30 |
| 0 | 0 | 0.06 | 0.94 | ≥ 10 | Year 1 |
| 0 | 0 | 0.265 | 0.735 | ≥ 10 | Year 5 |
| 0 | 0 | 0.51 | 0.49 | ≥ 10 | Year 10 |
| 0 | 0 | 0.658 | 0.342 | ≥ 10 | Year 15 |
| 0 | 0 | 0.796 | 0.204 | ≥ 10 | Year 20 |
| 0 | 0.338 | 0.617 | 0.045 | ≥ 10 | Year 30 |

| **Song Sparrow Density Gained (per acre)** | | | | | |
| --- | --- | --- | --- | --- | --- |
| **0 to < 1** | **1 to < 5** | **5 to < 10** | **≥ 10** | **RecoverSongSparrowDensity** | **PreRestSongSparrowDens** |
| 0.9 | 0.1 | 0 | 0 | 0 to < 5 | 0 to < 5 |
| 1 | 0 | 0 | 0 | 0 to < 5 | 5 to < 10 |
| 1 | 0 | 0 | 0 | 0 to < 5 | 10 to < 15 |
| 1 | 0 | 0 | 0 | 0 to < 5 | ≥ 15 |
| 0.3 | 0.6 | 0.1 | 0 | 5 to < 10 | 0 to < 5 |
| 0.5 | 0.5 | 0 | 0 | 5 to < 10 | 5 to < 10 |
| 0.9 | 0.1 | 0 | 0 | 5 to < 10 | 10 to < 15 |
| 0.9 | 0.1 | 0 | 0 | 5 to < 10 | ≥ 15 |
| 0.05 | 0.1 | 0.5 | 0.35 | 10 to < 15 | 0 to < 5 |
| 0.2 | 0.5 | 0.3 | 0 | 10 to < 15 | 5 to < 10 |
| 0.5 | 0.4 | 0.1 | 0 | 10 to < 15 | 10 to < 15 |
| 0.6 | 0.3 | 0.1 | 0 | 10 to < 15 | ≥ 15 |
| 0 | 0.1 | 0.3 | 0.6 | ≥ 15 | 0 to < 5 |
| 0 | 0.1 | 0.6 | 0.3 | ≥ 15 | 5 to < 10 |
| 0.05 | 0.4 | 0.35 | 0.2 | ≥ 15 | 10 to < 15 |
| 0.15 | 0.6 | 0.15 | 0.1 | ≥ 15 | ≥ 15 |

| **Recovering Song Sparrow Density (n/acre)** | | | | | |
| --- | --- | --- | --- | --- | --- |
| **0 to < 5** | **5 to <10** | **10 to < 15** | **≥ 15** | **RiparianHabitatQuality** | **TreeSwallowEgg** |
| 0.2 | 0.45 | 0.25 | 0.1 | Low | 0 to 0.3 |
| 0.4 | 0.35 | 0.2 | 0.05 | Low | 0.3 to 1.8 |
| 0.6 | 0.25 | 0.15 | 0 | Low | ≥ 1.8 |
| 0.1 | 0.2 | 0.4 | 0.3 | Medium | 0 to 0.3 |
| 0.2 | 0.35 | 0.3 | 0.15 | Medium | 0.3 to 1.8 |
| 0.4 | 0.4 | 0.2 | 0 | Medium | ≥ 1.8 |
| 0 | 0.05 | 0.4 | 0.55 | High | 0 to 0.3 |
| 0 | 0.4 | 0.35 | 0.25 | High | 0.3 to 1.8 |
| 0.1 | 0.6 | 0.3 | 0 | High | ≥ 1.8 |

| **Pre-Restoration Song Sparrow Density (n/acre)** | | | |
| --- | --- | --- | --- |
| **0 to < 5** | **5 to < 10** | **10 to < 15** | **≥ 15** |
| 0.3 | 0.5 | 0.2 | 0 |

| **Bird Egg-Equivalent Hg (µg/g)** | | | |
| --- | --- | --- | --- |
| **0 to < 0.3** | **0.3 to < 1.8** | **≥ 1.8** | **Time** |
| 0.5 | 0.3 | 0.2 | Year 1 |
| 0.6 | 0.25 | 0.15 | Year 5 |
| 0.7 | 0.2 | 0.1 | Year 10 |
| 0.8 | 0.15 | 0.05 | Year 15 |
| 0.9 | 0.1 | 0 | Year 20 |
| 0.95 | 0.05 | 0 | Year 30 |

| **Riparian Habitat Quality for Song Sparrows** | | | | | |
| --- | --- | --- | --- | --- | --- |
| **Low** | **Medium** | **High** | **RiparianPlantings** | **SoilTreatment** | **Year** |
| 1 | 0 | 0 | None | None | 1 |
| 0.97 | 0.03 | 0 | None | None | 5 |
| 0.95 | 0.05 | 0 | None | None | 10 |
| 0.9 | 0.1 | 0 | None | None | 15 |
| 0.9 | 0.1 | 0 | None | None | 20 |
| 0.9 | 0.1 | 0 | None | None | 30 |
| 0.95 | 0.05 | 0 | None | Topsoil | 1 |
| 0.9 | 0.1 | 0 | None | Topsoil | 5 |
| 0.85 | 0.15 | 0 | None | Topsoil | 10 |
| 0.75 | 0.25 | 0 | None | Topsoil | 15 |
| 0.75 | 0.25 | 0 | None | Topsoil | 20 |
| 0.75 | 0.25 | 0 | None | Topsoil | 30 |
| 0.95 | 0.05 | 0 | Willow | None | 1 |
| 0.7 | 0.2 | 0.1 | Willow | None | 5 |
| 0.2 | 0.45 | 0.35 | Willow | None | 10 |
| 0.1 | 0.3 | 0.6 | Willow | None | 15 |
| 0.05 | 0.1 | 0.85 | Willow | None | 20 |
| 0 | 0.1 | 0.9 | Willow | None | 30 |
| 0.9 | 0.1 | 0 | Willow | Topsoil | 1 |
| 0.55 | 0.25 | 0.2 | Willow | Topsoil | 5 |
| 0.1 | 0.4 | 0.5 | Willow | Topsoil | 10 |
| 0 | 0.2 | 0.8 | Willow | Topsoil | 15 |
| 0 | 0.1 | 0.9 | Willow | Topsoil | 20 |
| 0 | 0.05 | 0.95 | Willow | Topsoil | 30 |
| 1 | 0 | 0 | Herb. Seed | None | 1 |
| 0.8 | 0.2 | 0 | Herb. Seed | None | 5 |
| 0.6 | 0.3 | 0.1 | Herb. Seed | None | 10 |
| 0.4 | 0.4 | 0.2 | Herb. Seed | None | 15 |
| 0.2 | 0.5 | 0.3 | Herb. Seed | None | 20 |
| 0 | 0.6 | 0.4 | Herb. Seed | None | 30 |
| 0.95 | 0.05 | 0 | Herb. Seed | Topsoil | 1 |
| 0.65 | 0.25 | 0.1 | Herb. Seed | Topsoil | 5 |
| 0.45 | 0.3 | 0.25 | Herb. Seed | Topsoil | 10 |
| 0.25 | 0.4 | 0.35 | Herb. Seed | Topsoil | 15 |
| 0.1 | 0.35 | 0.55 | Herb. Seed | Topsoil | 20 |
| 0.05 | 0.3 | 0.65 | Herb. Seed | Topsoil | 30 |
| 0.95 | 0.05 | 0 | Herb. AND Willow | None | 1 |
| 0.65 | 0.25 | 0.1 | Herb. AND Willow | None | 5 |
| 0.1 | 0.5 | 0.4 | Herb. AND Willow | None | 10 |
| 0.05 | 0.25 | 0.7 | Herb. AND Willow | None | 15 |
| 0 | 0.1 | 0.9 | Herb. AND Willow | None | 20 |
| 0 | 0.05 | 0.95 | Herb. AND Willow | None | 30 |
| 0.9 | 0.1 | 0 | Herb. AND Willow | Topsoil | 1 |
| 0.45 | 0.3 | 0.25 | Herb. AND Willow | Topsoil | 5 |
| 0.05 | 0.35 | 0.6 | Herb. AND Willow | Topsoil | 10 |
| 0 | 0.05 | 0.95 | Herb. AND Willow | Topsoil | 20 |
| 0 | 0 | 1 | Herb. AND Willow | Topsoil | 30 |

**Table S1c.** Utility node values are determined by a combination of both the restoration treatment option (decision node) and inflation adjustment (nature node). There are two utility nodes in the Bird Mercury model: one for riparian planting treatment costs and one for soil amendment treatment costs.

| **Riparian Treatment Cost (acre)** | | |
| --- | --- | --- |
| **Dollar value** | **Riparian Planting Treatment** | **Inflation Adjustment (%)** |
| 0 | None | 0 to < 2.5 |
| 0 | None | 2.5 to < 5 |
| 0 | None | 5 to < 7.5 |
| 0 | None | 7.5 to 10 |
| $10250 | Willow Plant | 0 to < 2.5 |
| $10500 | Willow Plant | 2.5 to < 5 |
| $10750 | Willow Plant | 5 to < 7.5 |
| $11000 | Willow Plant | 7.5 to < 10 |
| $4100 | Herbaceous Seed | 0 to < 2.5 |
| $4200 | Herbaceous Seed | 2.5 to < 5 |
| $4300 | Herbaceous Seed | 5 to < 7.5 |
| $4400 | Herbaceous Seed | 7.5 to 10 |
| $14350 | Willow AND Herba | 0 to < 2.5 |
| $14700 | Willow AND Herba | 2.5 to < 5 |
| $15050 | Willow AND Herba | 5 to < 7.5 |
| $15400 | Willow AND Herba | 7.5 to 10 |

| **Soil Treatment Cost (acre)** | | |
| --- | --- | --- |
| **Dollar Value** | **Soil Treatment** | **Inflation Adjustment (%)** |
| 0 | None | 0 to < 2.5 |
| 0 | None | 2.5 to < 5 |
| 0 | None | 5 to < 7.5 |
| 0 | None | 7.5 to < 10 |
| $8200 | Topsoil Amendment | 0 to < 2.5 |
| $8400 | Topsoil Amendment | 2.5 to < 5 |
| $8600 | Topsoil Amendment | 5 to < 7.5 |
| $8800 | Topsoil Amendment | 7.5 to 10 |


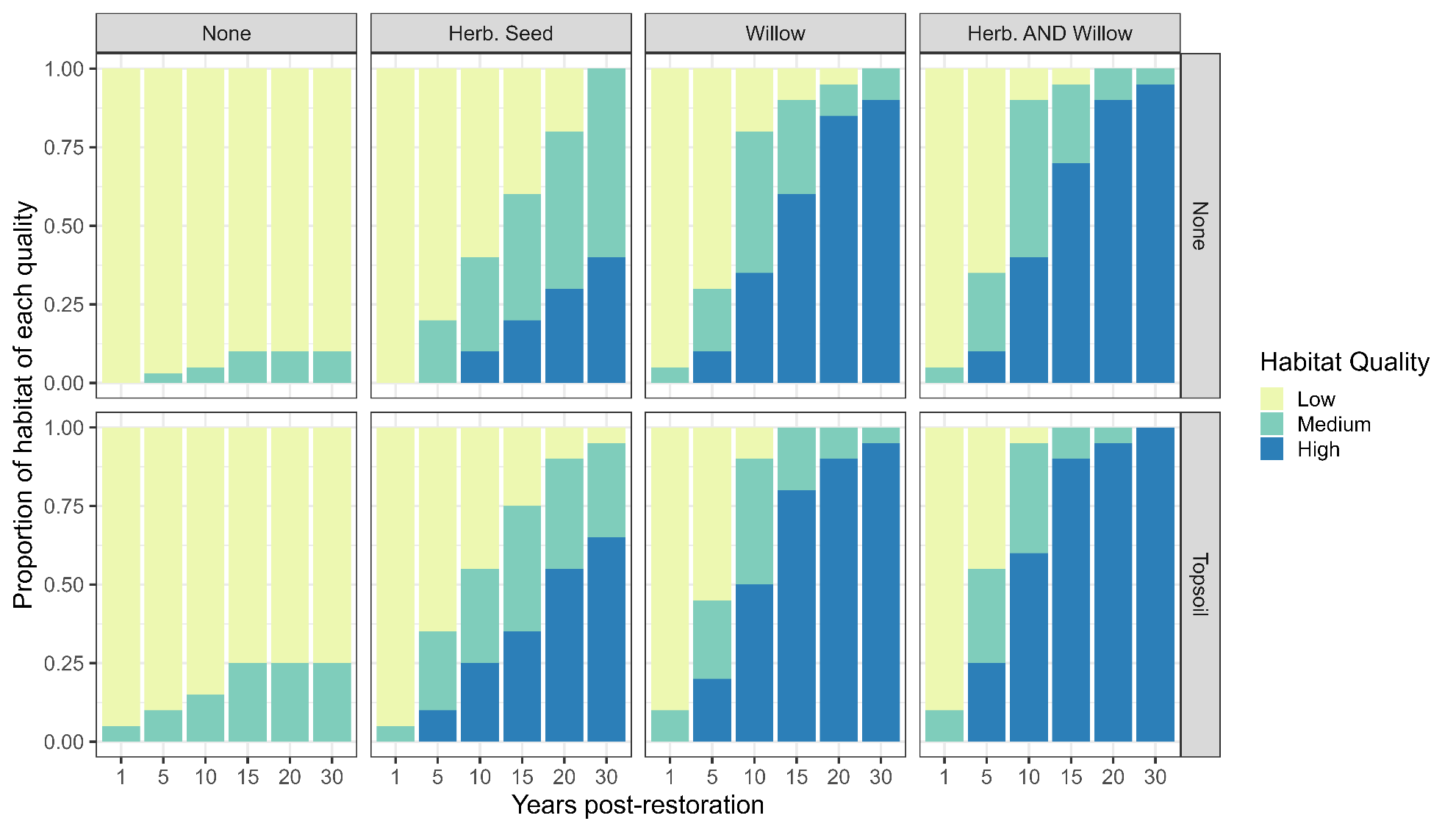


**Figure S1a.** Graphical representation of the conditional probability table structure for determining riparian habitat quality for Song Sparrows (*Melospiza melodia*) in the Bayesian decision network exploring restoration options. The restoration options include soil amendments (“None” and “Topsoil” amendments) x planting (“None”, “Herb. Seed” = herbaceous seeding, “Willow” = willow planting, and “Herb. AND Willow” = both herbaceous seeding and willow planting. We used scientific literature (see Methods) to determine the probability of riparian habitat being low, medium, or high quality for Song Sparrows occupying the affected area.


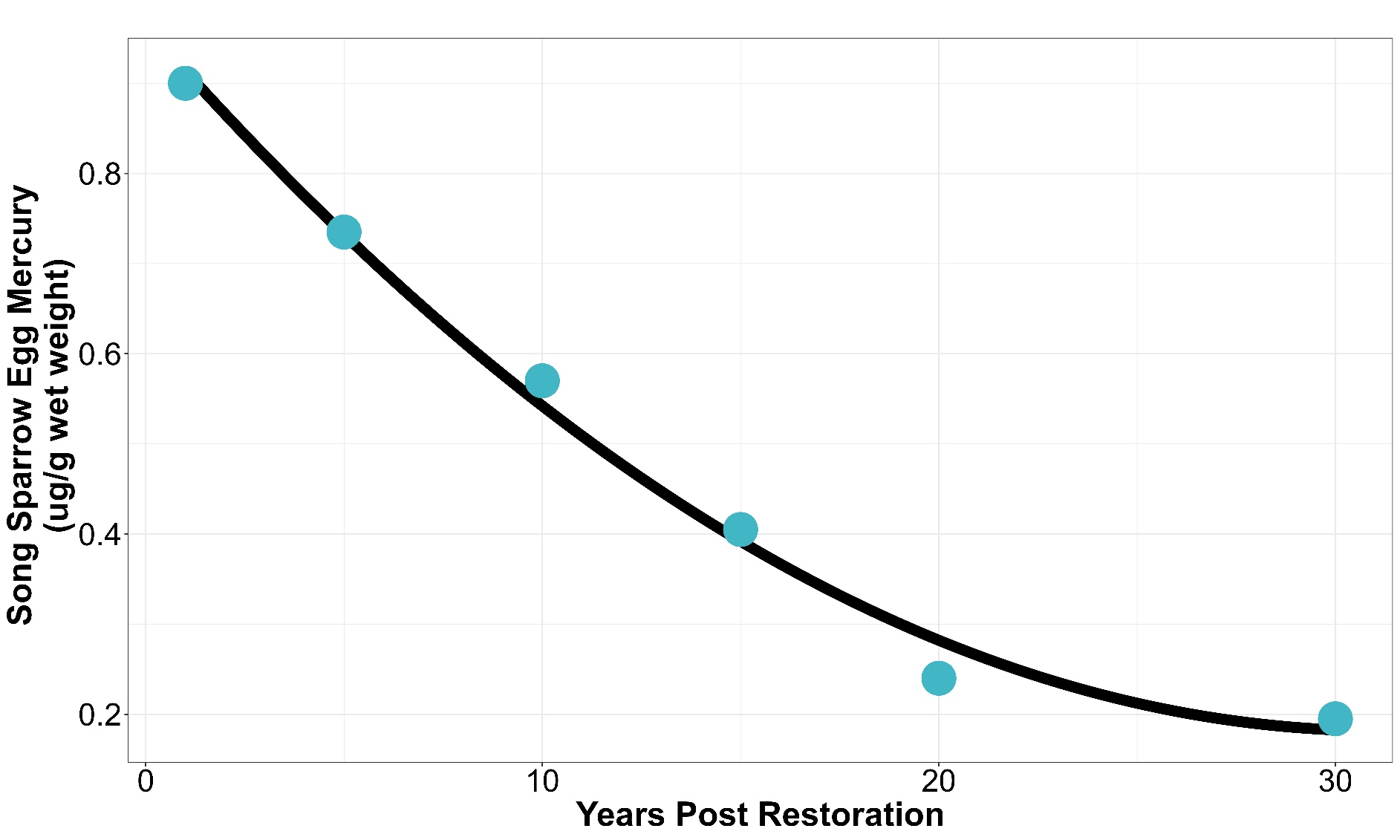


**Figure S1b**. Projected decrease in Song Sparrow egg mercury concentration following remediation and restoration

**Table S1d.** The parameters, parameter states, justification for inclusion, and references used to support the development of the Upper Arkansas River (UAR) Bayesian Network

| **Model Parameter** | **Parameter States** | **Justification** | **References** |
| --- | --- | --- | --- |
| **Observable measurements** | | | |
| **Weighted Usable Area (m^2^/m)** | 0 to < 1 | Weighted usable area (WUA) is a metric used to characterize stream habitat suitability for aquatic organisms. Two-dimensional hydrodynamic modeling was used by Richer et al. (2019) to estimate WUA for adult, juvenile, fry, and spawning brown trout (*Salmo trutta*) across a range of flows in the upper Arkansas River. We used the least-squares mean (LSmean) values of WUA reported by Richer et al. (2019). The WUA state values we chose bracket the lsmean WUA values observed in the UAR before and after habitat restoration. | Richer et al. 2019 |
|  | 1 to < 2 |  |  |
|  | ≥ 2 |  |  |
| **Average Habitat Heterogeneity (depth, velocity)** | 0 to < 0.5 | Habitat heterogeneity is a common goal for stream restoration projects, and it has been linked to increased biological diversity and ecological function. Richer et al. (2019) calculated habitat heterogeneity through calculations of the coefficient of variation (CV) for water depth and velocity, before and after restoration. | Palmer et al. 2010; Richer et al. 2019 |
|  | 0.5 to <0.75 |  |  |
|  | ≥ 0.75 |  |  |
| **Brown Trout Foraging Position  (n/m)** | 0 to < 0.5 | Foraging positions (FPs) are defined as locations within streams that trout may use to optimize the energy spent swimming, maintaining position, and searching for prey, while maximizing the encounter and consumption of prey resources. Richer et al. 2019 estimated foraging position using fish length, weight, focal point velocity, capture area velocity, foraging radius, and minimum depth. Foraging position is expressed as the number of fish per meter of stream. We bracket changes in FP before and after restoration in the UAR. | Fausch 1984; Richer et al. 2019 |
|  | 0.5 to < 1.0 |  |  |
|  | ≥ 1.0 |  |  |
| **Summary responses** | | | |
| **Stream Habitat Quality for Trout** | Low | An integrated stream habitat metric that combines variation of the three-habitat metrics: weighted usable area, average habitat heterogeneity of depth and velocity, and brown trout foraging position. These habitat metrics are often used as indicators of habitat quality for stream biota. | Richer et al. 2019 |
|  | Medium |  |  |
|  | High |  |  |
| **Biotic responses** | | | |
| **Benthic Macroinvertebrate Biomass  (mg/0.1 m^2^)** | 2000 to < 4000 | The biomass of benthic macroinvertebrates is reflective of stream productivity and an indicator of prey availability to trout. Kotalik et al. (2023) evaluated benthic biomass on the UAR through the habitat restoration reach, both before and after restoration. State ranges are parameterized after sites AR4C through AR4H, for UAR. | Kotalik et al. 2023 |
|  | 4000 to < 6000 |  |  |
|  | 6000 to 8000 |  |  |
| **Brown Trout Biomass (kg/ha)** | 50 to < 100 | Trout biomass is a standard metric to evaluate the health of fisheries. Biomass estimates were gathered from stream brown trout population surveys in the UAR using bank electrofishing and the two-pass removal method. Brown trout biomass state ranges bracket biomass observed on the UAR before and after habitat restoration. State ranges are parameterized after NRDA sites AR4C through AR4H, for UAR. | Richer et al. 2022; Kotalik et al. 2023 |
|  | 100 to <150 |  |  |
|  | 150 to 300 |  |  |
| **Time variable** | | | |
| **Time** | Year 0 | Year 0 represents pre-restoration conditions, bracketing the brown trout biomass observed after remediation completion, but before restoration was completed, from 2008 to 2014.  Year 0 represents pre-restoration  Year 5 represents observed responses  Years 10, 20, and 30 represent projections  Projections assume that brown trout require more time to respond to restoration compared to invertebrates or physical habitat metrics. | Richer et al. 2022; Kotalik et al. 2023 |
|  | Year 5 |  |  |
|  | Year 10 |  |  |
|  | Year 20 |  |  |
|  | Year 30 |  |  |
| **Restoration activities** | | | |
| **Instream Habitat Restoration** | Not Implement | Large-scale habitat restoration was conducted within a 4 km reach of the UAR from 2013 to 2014. Instream habitat restoration included the installation of structures such as log vanes, boulder clusters, and woody debris. “Not Implement” and “Implement” represent changes in habitat metrics before and after habitat restoration, respectively. | Richer et al. 2019; Richer et al. 2022; Kotalik et al. 2023 |
|  | Implement |  |  |
| **Bank Stabilization** | Not Implement | Large-scale habitat restoration was conducted within a 4 km reach of the UAR from 2013 to 2014. Bank stabilization was performed in specific stream reaches to reduce bank erosion. “Not Implement” and “Implement” represent changes in habitat metrics before and after habitat restoration, respectively. | Richer et al. 2019; Richer et al. 2022; Kotalik et al. 2023 |
|  | Implement |  |  |

**Table S1e.** Conditional probability tables for all nodes in the Upper Arkansas River Bayesian Decision Network used to determine the probability of Brown Trout (*Salmo trutta*) Biomass (kg/ha) before and after habitat restoration implementation. The posterior probability values, shown on a scale of [0,1], of the response node are listed in the first variable column set, and the respective state values of the parent nodes are listed in columns to the right of those.

| **Brown Trout Biomass (kg/ha)** | | | | | |
| --- | --- | --- | --- | --- | --- |
| **50 to < 100** | **100 to < 150** | **150 to 300** | **Stream Habitat Quality** | **Benthic Macroinvertebrate Biomass (mg/m^2^)** | **Projection** |
| 0.45 | 0.55 | 0 | Low | 2000 to < 4000 | Year 0 |
| 0.35 | 0.65 | 0 | Low | 2000 to < 4000 | Year 5 |
| 0.25 | 0.75 | 0 | Low | 2000 to < 4000 | Year 10 |
| 0.2 | 0.8 | 0 | Low | 2000 to < 4000 | Year 20 |
| 0.175 | 0.825 | 0 | Low | 2000 to < 4000 | Year 30 |
| 0.4 | 0.6 | 0 | Low | 4000 to < 6000 | Year 0 |
| 0.3 | 0.7 | 0 | Low | 4000 to < 6000 | Year 5 |
| 0.2 | 0.8 | 0 | Low | 4000 to < 6000 | Year 10 |
| 0.1 | 0.85 | 0.05 | Low | 4000 to < 6000 | Year 20 |
| 0.05 | 0.9 | 0.05 | Low | 4000 to < 6000 | Year 30 |
| 0.3 | 0.7 | 0 | Low | 6000 to 8000 | Year 0 |
| 0.2 | 0.8 | 0 | Low | 6000 to 8000 | Year 5 |
| 0.1 | 0.85 | 0.05 | Low | 6000 to 8000 | Year 10 |
| 0.05 | 0.85 | 0.1 | Low | 6000 to 8000 | Year 20 |
| 0.025 | 0.875 | 0.1 | Low | 6000 to 8000 | Year 30 |
| 0.4 | 0.6 | 0 | Medium | 2000 to < 4000 | Year 0 |
| 0.3 | 0.65 | 0.05 | Medium | 2000 to < 4000 | Year 5 |
| 0.2 | 0.7 | 0.1 | Medium | 2000 to < 4000 | Year 10 |
| 0.15 | 0.7 | 0.15 | Medium | 2000 to < 4000 | Year 20 |
| 0.1 | 0.75 | 0.15 | Medium | 2000 to < 4000 | Year 30 |
| 0.3 | 0.7 | 0 | Medium | 4000 to < 6000 | Year 0 |
| 0.2 | 0.7 | 0.1 | Medium | 4000 to < 6000 | Year 5 |
| 0.1 | 0.75 | 0.15 | Medium | 4000 to < 6000 | Year 10 |
| 0.05 | 0.75 | 0.2 | Medium | 4000 to < 6000 | Year 20 |
| 0.025 | 0.75 | 0.225 | Medium | 4000 to < 6000 | Year 30 |
| 0.2 | 0.8 | 0 | Medium | 6000 to 8000 | Year 0 |
| 0.1 | 0.8 | 0.1 | Medium | 6000 to 8000 | Year 5 |
| 0 | 0.8 | 0.2 | Medium | 6000 to 8000 | Year 10 |
| 0 | 0.75 | 0.25 | Medium | 6000 to 8000 | Year 20 |
| 0 | 0.725 | 0.27.5 | Medium | 6000 to 8000 | Year 30 |
| 0.3 | 0.7 | 0 | High | 2000 to < 4000 | Year 0 |
| 0.2 | 0.75 | 0.05 | High | 2000 to < 4000 | Year 5 |
| 0.1 | 0.75 | 0.15 | High | 2000 to < 4000 | Year 10 |
| 0.05 | 0.75 | 0.2 | High | 2000 to < 4000 | Year 20 |
| 0.025 | 0.75 | 0.225 | High | 2000 to < 4000 | Year 30 |
| 0.2 | 0.7 | 0.1 | High | 4000 to < 6000 | Year 0 |
| 0.1 | 0.7 | 0.2 | High | 4000 to < 6000 | Year 5 |
| 0.05 | 0.7 | 0.25 | High | 4000 to < 6000 | Year 10 |
| 0 | 0.7 | 0.3 | High | 4000 to < 6000 | Year 20 |
| 0 | 0.675 | 0.325 | High | 4000 to < 6000 | Year 30 |
| 0 | 0.8 | 0.2 | High | 6000 to 8000 | Year 0 |
| 0 | 0.7 | 0.3 | High | 6000 to 8000 | Year 5 |
| 0 | 0.6 | 0.4 | High | 6000 to 8000 | Year 10 |
| 0 | 0.55 | 0.45 | High | 6000 to 8000 | Year 20 |
| 0 | 0.575 | 0.425 | High | 6000 to 8000 | Year 30 |

| **Benthic Macroinvertebrate Biomass (mg/m^2^)** | | | |
| --- | --- | --- | --- |
| **2000 to < 4000** | **4000 to < 6000** | **6000 to 8000** | **Stream Habitat Quality** |
| 0.3 | 0.6 | 0.1 | Low |
| 0.25 | 0.5 | 0.25 | Medium |
| 0.1 | 0.3 | 0.6 | High |

| **Stream Habitat Quality** | | | | | |
| --- | --- | --- | --- | --- | --- |
| **Low** | **Medium** | **High** | **Weighted Usable Area (m^2^/m)** | **Average Habitat Heterogeneity (CV)** | **Brown Trout Foraging Position (n/m)** |
| 0.7 | 0.3 | 0 | 0 to < 1 | 0 to < 0.5 | 0 to < 0.5 |
| 0.65 | 0.35 | 0 | 0 to < 1 | 0 to < 0.5 | 0.5 to < 1 |
| 0.6 | 0.4 | 0 | 0 to < 1 | 0 to < 0.5 | ≥ 1 |
| 0.65 | 0.35 | 0 | 0 to < 1 | 0.5 to < 0.75 | 0 to < 0.5 |
| 0.6 | 0.4 | 0 | 0 to < 1 | 0.5 to < 0.75 | 0.5 to < 1 |
| 0.55 | 0.45 | 0 | 0 to < 1 | 0.5 to < 0.75 | ≥ 1 |
| 0.6 | 0.35 | 0.05 | 0 to < 1 | ≥ 0.75 | 0 to < 0.5 |
| 0.55 | 0.35 | 0.1 | 0 to < 1 | ≥ 0.75 | 0.5 to < 1 |
| 0.45 | 0.4 | 0.15 | 0 to < 1 | ≥ 0.75 | ≥ 1 |
| 0.5 | 0.5 | 0 | 1 to < 2 | 0 to < 0.5 | 0 to < 0.5 |
| 0.4 | 0.6 | 0 | 1 to < 2 | 0 to < 0.5 | 0.5 to < 1 |
| 0.5 | 0.5 | 0 | 1 to < 2 | 0 to < 0.5 | ≥ 1 |
| 0.4 | 0.6 | 0 | 1 to < 2 | 0.5 to < 0.75 | 0 to < 0.5 |
| 0.5 | 0.5 | 0 | 1 to < 2 | 0.5 to < 0.75 | 0.5 to < 1 |
| 0.3 | 0.6 | 0.1 | 1 to < 2 | 0.5 to < 0.75 | ≥ 1 |
| 0.5 | 0.5 | 0 | 1 to < 2 | ≥ 0.75 | 0 to < 0.5 |
| 0.3 | 0.6 | 0.1 | 1 to < 2 | ≥ 0.75 | 0.5 to < 1 |
| 0 | 0.8 | 0.2 | 1 to < 2 | ≥ 0.75 | ≥ 1 |
| 0.1 | 0.2 | 0.7 | ≥ 2 | 0 to < 0.5 | 0 to < 0.5 |
| 0.05 | 0.25 | 0.7 | ≥ 2 | 0 to < 0.5 | 0.5 to < 1 |
| 0 | 0.15 | 0.85 | ≥ 2 | 0 to < 0.5 | ≥ 1 |
| 0.05 | 0.25 | 0.7 | ≥ 2 | 0.5 to < 0.75 | 0 to < 0.5 |
| 0 | 0.2 | 0.8 | ≥ 2 | 0.5 to < 0.75 | 0.5 to < 1 |
| 0 | 0.1 | 0.9 | ≥ 2 | 0.5 to < 0.75 | ≥ 1 |
| 0 | 0.15 | 0.85 | ≥ 2 | ≥ 0.75 | 0 to < 0.5 |
| 0 | 0.1 | 0.9 | ≥ 2 | ≥ 0.75 | 0.5 to < 1 |
| 0 | 0.05 | 0.95 | ≥ 2 | ≥ 0.75 | ≥ 1 |

| **Weighted Usable Area (m^2^/m)** | | | | |
| --- | --- | --- | --- | --- |
| **0 to < 1** | **1 to < 2** | **≥ 2** | **Instream Habitat Restoration** | **Bank Stabilization** |
| 0.2 | 0.8 | 0 | Not implement | Not implement |
| 0.15 | 0.75 | 0.1 | Not implement | Implement |
| 0.15 | 0.75 | 0.1 | Implement | Not implement |
| 0.1 | 0.7 | 0.2 | Implement | Implement |

| **Brown Trout Foraging Position (n/m)** | | | |
| --- | --- | --- | --- |
| **0 to < 0.5** | **0.5 to < 1** | **≥ 1** | **Instream Habitat Restoration** |
| 0.5 | 0.4 | 0.1 | Not implement |
| 0.25 | 0.5 | 0.25 | Implement |

| **Average Habitat Heterogeneity (CV)** | | | | |
| --- | --- | --- | --- | --- |
| **0 to < 0.5** | **0.5 to < 0.75** | **≥ 0.75** | **Instream Habitat Restoration** | **Bank Stabilization** |
| 0.3 | 0.65 | 0.05 | Not implement | Not implement |
| 0.1 | 0.7 | 0.2 | Not implement | Implement |
| 0.1 | 0.8 | 0.1 | Implement | Not implement |
| 0.05 | 0.7 | 0.25 | Implement | Implement |

**SI-2**

**Comparison of Song Sparrow Model and Brown Trout Model for model outcome sensitivity and parameter influence**

**Sensitivity Analyses**

The Song Sparrow and Brown Trout Bayesian decision network models were each analyzed for sensitivity of their final outcome nodes to other nodes in the network. Sensitivity analysis is conducted on the models set to their default probability states, that is, with no specific inputs or state conditions specified other than the prior values of the model's conditional probability table settings.

To conduct sensitivity analyses, the decision and utility nodes were first removed from the models as per procedures with the Netica Bayesian network modeling program, as sensitivity analysis in that program cannot be conducted on models with such nodes included. Sensitivity analysis was then conducted on the models to reveal the degree to which the remaining nodes influence the final outcome nodes in each model. This essentially reflects the causal structure and the underlying probability parameters (conditional probability table values) of the models.

The sensitivity analysis results presented here, for each node in the analysis, constitute estimates of variance reduction, the percentage of total variance reduction, and the *variance of belie*fs. As used in Netica, variance reduction is the expected reduction in the variation of the expected real value of the output variable with a specified number of states, due to the value of an input variable with its specified number of states. Variance reduction applies to continuous-value variables, as used in the models tested here, and their values span [0, infinity]. Variance of beliefs (also termed quadratic score) is defined in Netica as the expected change squared of the beliefs of the output variable, taken over all of its states, due to the values of an input variable. See Marcot (2012) for formulae used.^[[1]](#footnote-2)^

**Influence Run Analysis**

In contrast to model sensitivity analysis, influence run analysis consists of setting specified nodes to their extreme state conditions and recording the probability results of a specified output node in the model. This helps determine not the underlying sensitivity of the output node, but rather the potential ranges of influence of other nodes on the output node.

For this analysis, we evaluated the influence of the lowest level of the biotic response nodes to the final output nodes of the two models. We set the states of those biotic response nodes individually to their lowest and highest states, and recorded the state probability structure, including the expected value, of the final output nodes. We ran the analyses using the batch mode functions in Netica. We present results as the overall range of responses of the final output nodes to each influence setting. For more information on influence run methods and interpretations, see Morgan and Henrion (1990) and Marcot (2012), with applications in Atwood et al. (2016).

**Song Sparrow Model Sensitivity Analysis**


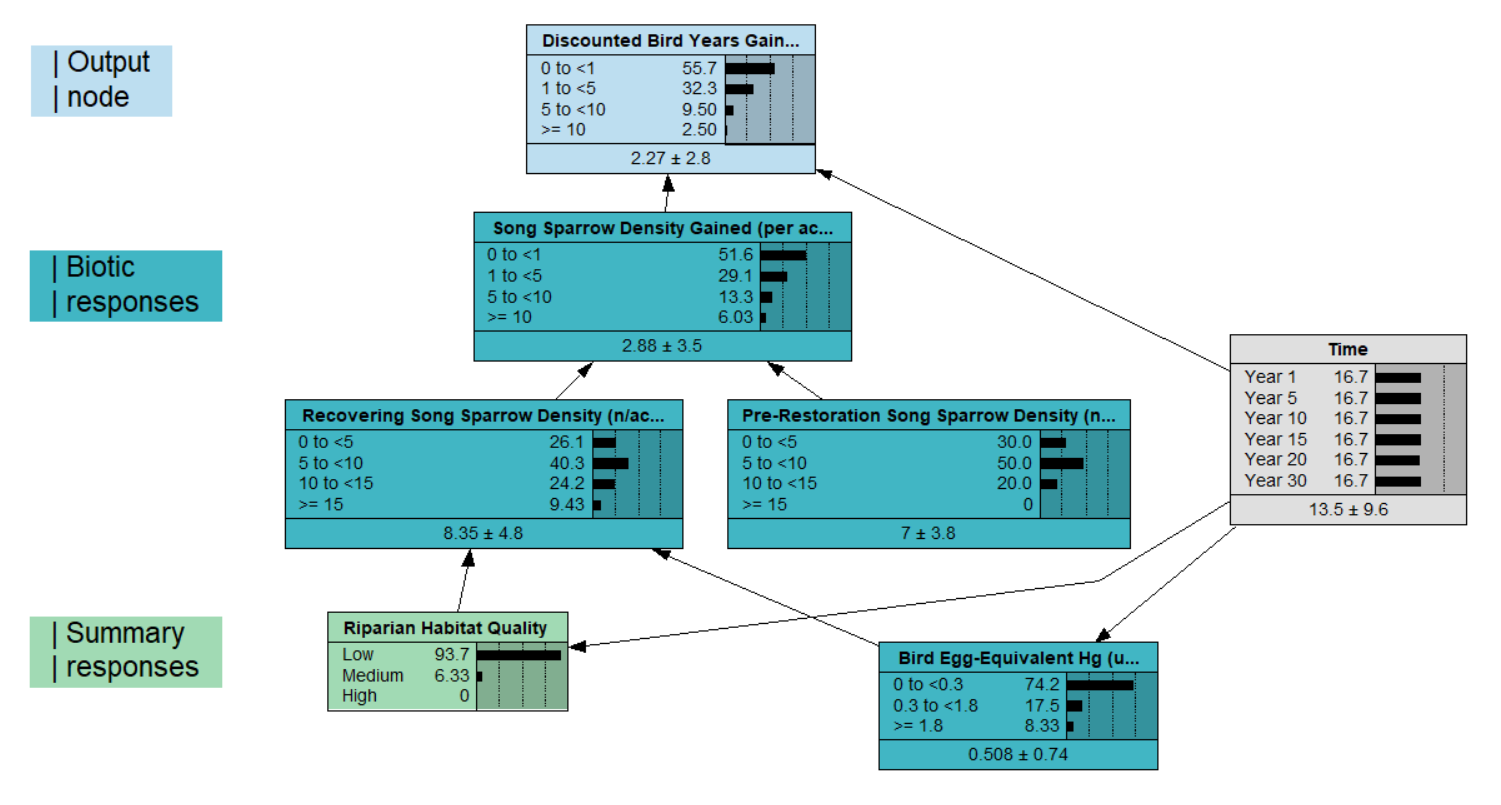


**Figure S2a.** Song Sparrow (*Melospiza melodia*) Bayesian network model as tested for sensitivity of the final outcome node (Discounted Bird Years Gained) to other nodes in the model, with decision and utility nodes removed for computation. For the full model, see main text **Figure 2**.

**Table S2a**. Sensitivity analysis results of the Song Sparrow (*Melospiza melodia*) Bayesian network model, showing sensitivity of the final outcome node Discounted Bird Years Gained to each other node in the model (sans decision and utility nodes).

Variance Percent Variance of

Node Reduction Reduction Beliefs

------------------------------------------------ ------------- -------------- ---------------

Discounted Bird Years Gained 9.44 100 0.3587754

Song Sparrow Density Gained 6.65 70.5 0.2010688

Recovering Song Sparrow Density 3.19 33.9 0.0596888

Pre-Restoration Song Sparrow Density 0.60 6.35 0.0114139

Bird Egg-Equivalent mercury 0.08 0.895 0.0040673

Time 0.07 0.796 0.0006232

Riparian Habitat Quality 0.05 0.594 0.0011496

The Song Sparrow Model sensitivity analysis, above, suggests that the output node Discounted Bird Years Gained has decreasing sensitivity to the biotic response nodes in order of the number of links distance in the network. This overall pattern of decreasing sensitivity to network distance is evident with values of variance reduction, percent reduction, and variance of beliefs. In other words, the output node Discounted Bird Years Gained is most sensitive to Song Sparrow Density Gained, which is an immediate parent node, second-most sensitive to Recovering Song Sparrow Density and to Pre-Restoration Song Sparrow Density, both of which are two links distant, etc. The anomaly is the low sensitivity to Time, but that in turn is because Time is a parent node to the lowermost biotic response nodes, Bird Egg-Equivalent Hg, and Riparian Habitat Quality.

In general, the sensitivity structure of the Song Sparrow Model follows generally expected patterns of decreasing sensitivity of the output node to further network distance to other nodes in the network. However, this does not mean that distant nodes can be disregarded in the causal structure, nor that link distance from an output node necessarily always determines relative influence effects.

**Brown Trout Model Sensitivity Analysis**


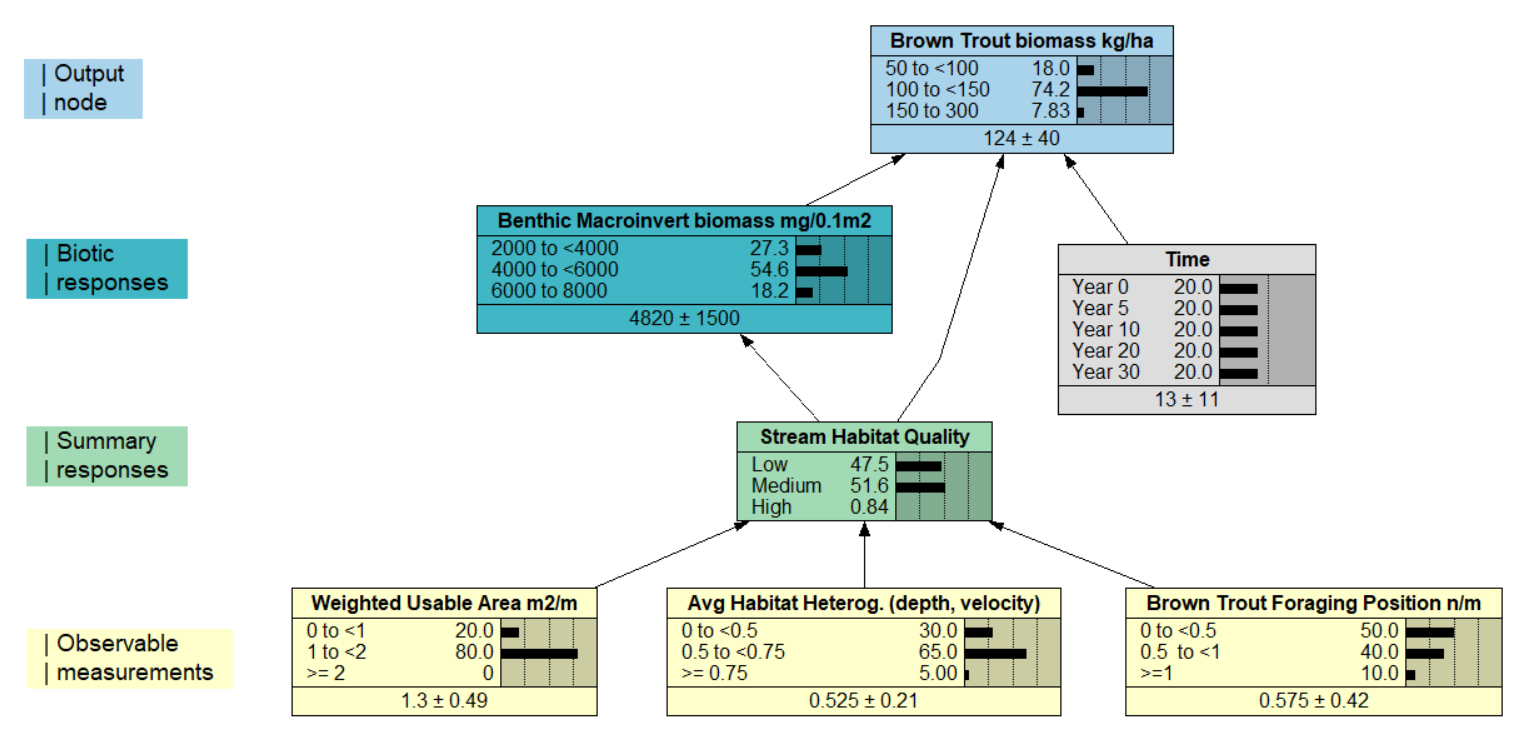


**Figure S2b.** Brown Trout (*Salmo trutta*) Bayesian network model as tested for sensitivity of the final outcome node (Brown Trout Biomass) to other nodes in the model, with decision and utility nodes removed for computation. For the full model, see main text **Figure 3**. Macroinvert = macroinvertebrate; Avg = average; Heterog. = heterogeneity.

**Table S2b**. Sensitivity analysis results of the Brown Trout Bayesian network model, showing sensitivity of the final outcome node Brown Trout (*Salmo trutta*) Biomass to all other nodes in the model (sans decision and utility nodes).

Variance Percent Variance of

Node Reduction Reduction Beliefs

------------------------------------------------ ------------- -------------- ---------------

Brown Trout Biomass 1570.00 100 0.2367329

Time 105.30 6.71 0.0050063

Stream Habitat Quality 67.94 4.33 0.0008659

Benthic Macroinvertebrate Biomass 38.29 2.44 0.0013176

Weighted Usable Area 1.63 0.104 0.0000190

Brown Trout Foraging Position 0.70 0.0451 0.0000095

Average Habitat Heterogeneity 0.36 0.0234 0.0000049

The above results of the Brown Trout model sensitivity analysis generally reflect the pattern of the outcome node Brown Trout Biomass having decreasing sensitivity to nodes further separated in the network structure.

One interesting comparison with the Song Sparrow model sensitivity analysis is the position of the Time node, which contributes a much higher sensitivity effect in the Brown Trout model. However, this is explained by the differences in the network structures of the two models. In the Brown Trout model, the time node links directly and only to the outcome node Brown Trout Biomass, whereas in the Song Sparrow model, the time node also informs the two biotic response nodes that are lowest in the network structure.

**Song Sparrow (*Melospiza melodia*) Model Influence Run Analysis**

We analyzed the influence on the final outcome node (Discounted Bird Years Gained) from setting extreme states individually of two biotic response nodes (Riparian Habitat Quality, Bird Egg-Equivalent Hg) and one affecter node (Time). We gauged influence by comparing the expected values of the outcome node and of the probability of the best state of the outcome node (>= 10 discounted bird years gained).


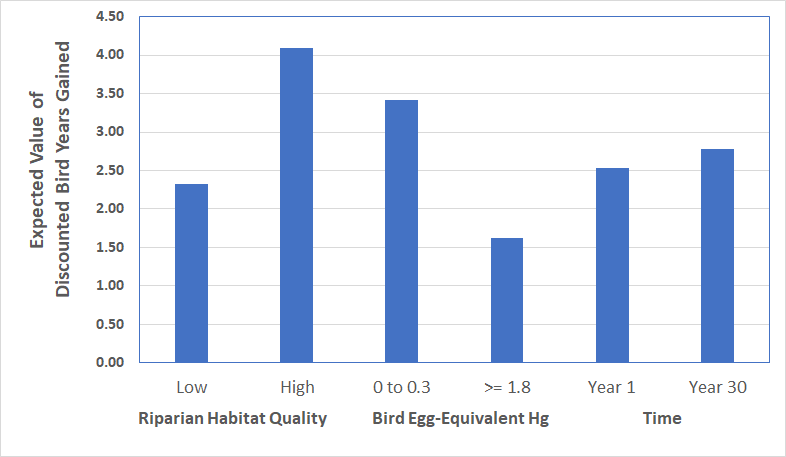


**Figure S2c.** Results of influence run analyses on Song Sparrow (*Melospiza melodia*) expected value of Discounted Bird Years Gained, by setting the two lowermost biotic response nodes and the Time node to their extreme (lowest and highest) states. Higher expected values denote more favorable outcomes for Song Sparrow.


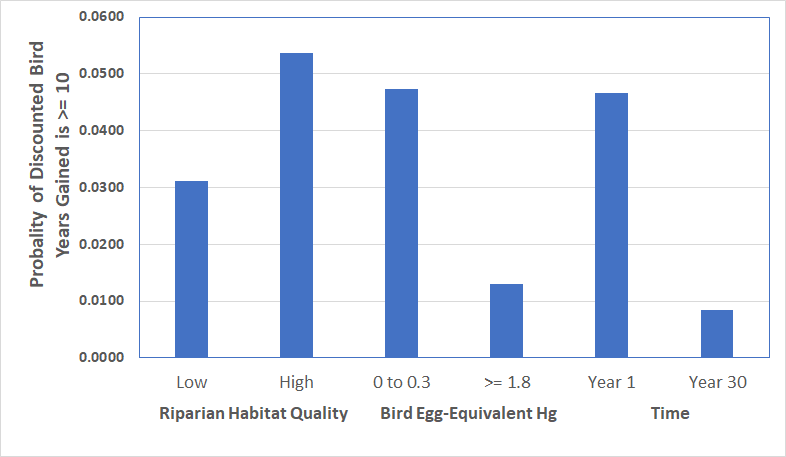


**Figure S2d.** Results of influence run analyses on Song Sparrow (*Melospiza melodia*) probability of the highest state of Discounted Bird Years Gained (>= 10), by setting the two lowermost biotic response nodes and the Time node to their extreme (lowest and highest) states. Higher probability values denote more favorable outcomes for Song Sparrow.

**Table S2c.** Range of influence results on Song Sparrow (*Melospiza melodia*) Discounted Bird Years Gained (DBYG) from setting the two lowermost biotic response nodes and the Time node to their extreme (lowest and highest) states. Values are the differences of expected values and best DBYG state probability values shown in **Figure S2c** and **Figure S2d**, above.

|  | Range of influence | |
| --- | --- | --- |
|  | DBYG  expected value | Highest DBYG state |
|  |  |  |
| Riparian Habitat Quality | 1.77 | 0.0225 |
| Bird Egg-Equivalent Hg | 1.79 | 0.0343 |
| Time | 0.24 | -0.0383 |

The results above suggest a significant and nearly equivalent influence from the two biotic response nodes on the expected value and the best outcome state of Discounted Bird Years Gained. Results show that Time has a low influence on the expected value of Discounted Bird Years Gained. The major and negative influence of Time on the probability of the best outcome state for Discounted Bird Years Gained is explained by how, in the Song Sparrow model, the Year 1 state of the Time node results in a dominant probability of the lowest state (0 to 1) of Discounted Bird Years Gained, and the Year 30 state of the Time node shifts most of the probability of the highest state of Discounted Bird Years Gained to its second state (1 to 5). Regardless of this quirk, note that the probability values of the highest state of Discounted Bird Years Gained remain very low with all possible outcomes (i.e., <1 percent probability).

**Brown Trout (*Salmo trutta*) Model Influence Run Analysis**

We analyzed the influence on the final outcome node (Brown Trout Biomass) from setting extreme states individually of three biotic response nodes (Weighted Usable Area, Average Habitat Heterogeneity, and Brown Trout Foraging Position). Time, as used in the Song Sparrow influence run analysis, was included here because it directly links only to the outcome node, whereas the influence run analyses were structured to track effects of the biotic response variables. We gauged influence by comparing the expected values of the outcome node and of the probability of the best state of the outcome node (150 to 300 kg/ha).


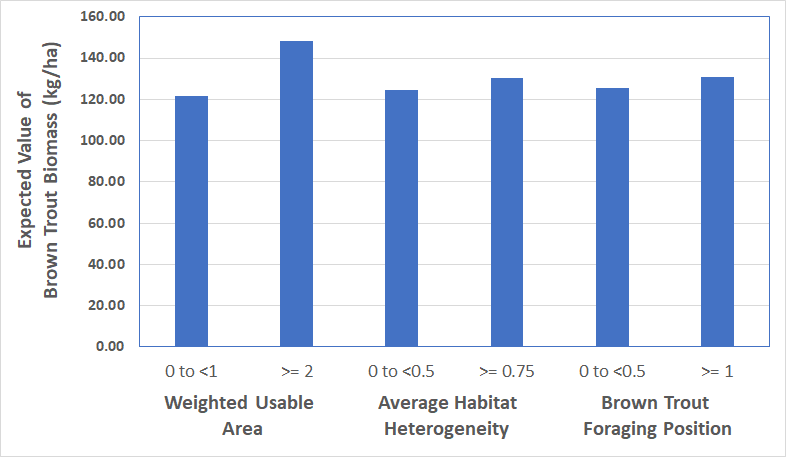


**Figure S2e.** Results of influence run analyses on the expected value of Brown Trout (*Salmo trutta*) Biomass, with the three lowermost biotic response nodes set to their extreme states (lowest and highest). Higher expected values denote more favorable outcomes for Brown Trout.


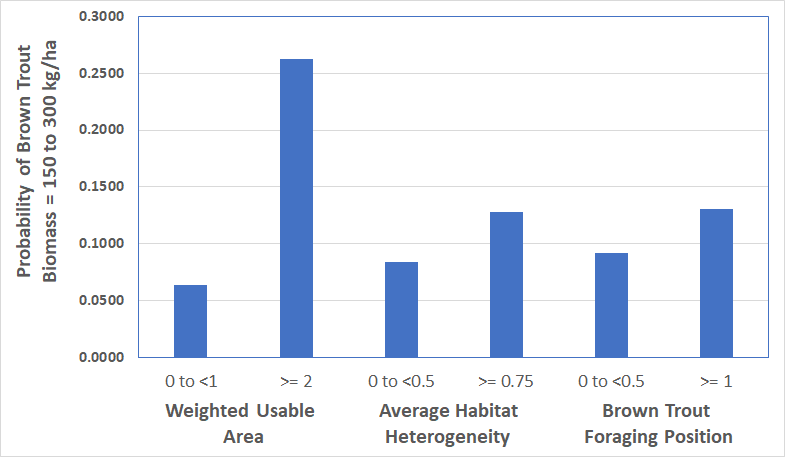


**Figure S2f.** Results of influence run analyses on the probability of the highest state of Brown Trout (*Salmo trutta*) Biomass (150 to 300 kg/ha), by setting the three lowermost biotic response nodes to their extreme (lowest and highest) states. Higher probability values denote more favorable outcomes for Brown Trout.

**Table S2d.** Range of influence results on Brown Trout (*Salmo trutta*) Biomass from setting the three lowermost biotic response nodes to their extreme (lowest and highest) states. Values are the differences of expected values and best Brown Trout Biomass state probability values shown in **Figure S2e** and **Figure S2f**, above.

|  | Range of influence | |
| --- | --- | --- |
|  | Brown Trout Biomass expected value | Best Brown Trout Biomass state |
|  |  |  |
| Weighted Usable Area | 26.63 | 0.1991 |
| Average Habitat Heterogeneity | 5.92 | 0.0441 |
| Brown Trout Foraging Position | 5.20 | 0.0387 |

Results, above, suggest relatively similar influence on the expected value of Brown Trout Biomass from Average Habitat Heterogeneity and Brown Trout Foraging Position, but far greater influence from Weighted Usable Area. The influence of Weighted Usable Area is accounted for in the model because its highest state (>= 2 m^2^/m) causes the dominant probability of the "High" state in Stream Habitat Quality (with all else set to their normative prior probability conditions), which in turn propagates further through the network as a dominant probability of the highest state of Benthic Macroinvertebrate Biomass. This is a lesson in how the influence of one state in a lower-level node in a network can have a profound impact on variables further along a network pathway.

**References**

Ackerman, J.T., Peterson, S.H., Herzog, M.P., Yee, J.L., 2024. Methylmercury effects on birds: A review, meta‐analysis, and development of toxicity reference values for injury assessment based on tissue residues and diet. Environmental Toxicology and Chemistry 43, 1195-1241.

Atwood, T. C., B. G. Marcot, D. C. Douglas, S. C. Amstrup, K. D. Rode, G. M. Durner, and J. F. Bromaghin. 2016. Forecasting the relative influence of anthropogenic stressors on polar bears. Ecosphere 7(6): <https://doi.org/10.1002/ecs2.1370>.’’

Baker, M., Domanski, A., Hollweg, T., Murray, J., Lane, D., Skrabis, K., Taylor, R., Moore, T., DiPinto, L., 2020. Restoration scaling approaches to addressing ecological injury: the habitat-based resource equivalency method. Environmental Management 65, 161-177.

Campos, B.R., Burnett, R.D., Loffland, H.L., Siegel, R.B., 2020. Bird response to hydrologic restoration of montane riparian meadows. Restoration Ecology 28, 1262-1272.

Dybala, K.E., Engilis, A., Trochet, J.A., Engilis, I.E., Truan, M.L., 2018. Evaluating riparian restoration success: long-term responses of the breeding bird community in California’s lower Putah Creek watershed. Ecological Restoration 36, 76-85.

Eckley, C.S., Gilmour, C.C., Janssen, S., Luxton, T.P., Randall, P.M., Whalin, L., Austin, C., 2020. The assessment and remediation of mercury contaminated sites: A review of current approaches. Science of the Total Environment 707, 136031.

Fausch, K.D., 1984. Profitable stream positions for salmonids: relating specific growth rate to net energy gain. Canadian journal of zoology 62, 441-451.

Gardali, T., Scoggin, S.E., Geupel, G.R., 1999. Songbird use of Redwood and Lagunitas creeks: management and restoration recommendations. Point Reyes Bird Observatory report to the Golden Gate National Recreation Area. Stinson Beach, California, USA.https://www.researchgate.net/profile/Geoffrey-Geupel/publication/252230478_SONGBIRD_USE_OF_REDWOOD_AND_LAGUNITAS_CREEKS_MANAGEMENT_AND_RESTORATION_RECOMMENDATIONS/links/004635388d2890690e000000/SONGBIRD-USE-OF-REDWOOD-AND-LAGUNITAS-CREEKS-MANAGEMENT-AND-RESTORATION-RECOMMENDATIONS.pdf

Germain, R.R., Schuster, R., Tarwater, C.E., Hochachka, W.M., Arcese, P., 2018. Adult survival and reproductive rate are linked to habitat preference in territorial, year‐round resident Song Sparrows Melospiza melodia. Ibis 160, 568-581.

González-Sargas, E., Meehan, T.D., Hinojosa-Huerta, O., Villagomez-Palma, S., Calvo-Fonseca, A., Dodge, C., Gómez-Sapiens, M., Shafroth, P.B., 2024. Bird community response to one decade of riparian restoration along the Colorado River delta in Mexico. Ecological Engineering 205, 107291.

Horsch, E., Phaneuf, D., Giguere, C., Murray, J., Duff, C., Kroninger, C., 2023. Discounting in natural resource damage assessment. Journal of Benefit-Cost Analysis 14, 141-161.

Julius, B., 1999. Discounting and the treatment of uncertainty in natural resource damage assessment. *Technical paper* 99 (1).

Kotalik, C.J., Wolff, B.A., Pomeranz, J.P., Richer, E.E., Clements, W.H., 2023. Bioenergetic responses of a stream food web to habitat restoration: interactions between Brown trout and invertebrate prey resources. Restoration Ecology 31, e13908.

Marcot, B. G. 2012. Metrics for evaluating performance and uncertainty of Bayesian network models. Ecological Modelling 230:50-62. <https://doi.org/10.1016/j.ecolmodel.2012.01.013>.

Morgan, M. G., and M. Henrion. 1990. Uncertainty: a guide to dealing with uncertainty in quantitative risk and policy analysis. Cambridge University Press, New York. 344 pp.

Palmer, Margaret A., Holly L. Menninger, and Emily Bernhardt. "River restoration, habitat heterogeneity and biodiversity: a failure of theory or practice?." Freshwater biology 55 (2010): 205-222.

Shanahan, S., Nelson, S., Van Dooremolen, D., Eckberg, J., 2011. Restoring habitat for riparian birds in the lower Colorado River watershed: An example from the Las Vegas Wash, Nevada. Journal of Arid Environments 75, 1182-1190.

Stephens, J.L., Rockwell, S.M., 2019. Short-term riparian restoration success measured by territory density and reproductive success of three songbirds along the Trinity River, California. The Condor 121, duz043.

Watts, B.D., 1990. Cover use and predator-related mortality in song and savannah sparrows. The Auk 107, 775-778.

Zentner, J., Glaspy, J., Schenk, D., 2003. Wetland and riparian woodland restoration costs. Ecological Restoration 21, 166-173.

1. Also see: <https://www.norsys.com/WebHelp/NETICA.htm#NETICA/X_Scoring_Rule_Results.htm>. [↑](#footnote-ref-2)
